# RNA-Seq reveals the existence of a *CDKN1C-E2F1-TP53* axis that is altered in human T-cell lymphoblastic lymphomas

**DOI:** 10.1186/s12885-018-4304-y

**Published:** 2018-04-16

**Authors:** Pilar López-Nieva, Pablo Fernández-Navarro, Concepción Vaquero-Lorenzo, María Villa-Morales, Osvaldo Graña-Castro, María Ángeles Cobos-Fernández, José Luis López-Lorenzo, Pilar Llamas, Laura González-Sanchez, Isabel Sastre, Marina Pollan, Marcos Malumbres, Javier Santos, José Fernández-Piqueras

**Affiliations:** 1 Department of Cellular Biology and Immunology, Severo Ochoa Molecular Biology Center (CBMSO), CSIC-Madrid Autonomous University, 28049 Madrid, Spain; 2 Institute of Health Research, Jiménez Díaz Foundation, Madrid, Spain; 3 Consortium for Biomedical Research in Rare Diseases (CIBERER), Carlos III Institute of Health, Madrid, Spain; 4 Cancer and Environmental Epidemiology Unit, National Center for Epidemiology, Carlos III Institute of Health, Madrid, Spain; 5 Consortium for Biomedical Research in Epidemiology and Public Health (CIBERESP), Madrid, Spain; 6 Bioinformatics Unit, Structural Biology and Biocomputing Programme, Spanish National Cancer Research Center (CNIO), Madrid, Spain; 7 Cell Division and Cancer Group, Molecular Oncology Programme, Spanish National Cancer Research Centre (CNIO), Madrid, Spain

**Keywords:** T-cell lymphoblastic lymphoma, *CDKN1C-E2F1-TP53* deregulation, Promoter hypermethylation, Deregulation of miRNAs

## Abstract

**Background:**

Precursor T-cell lymphoblastic lymphomas (T-LBL) are rare aggressive hematological malignancies that mainly develop in children. As in other cancers, the loss of cell cycle control plays a prominent role in the pathogenesis in these malignancies that is primarily attributed to loss of *CDKN2A* (encoding protein p16INK4A). However, the impact of the deregulation of other genes such as *CDKN1C*, *E2F1, and TP53* remains to be clarified. Interestingly, experiments in mouse models have proven that conditional T-cell specific deletion of Cdkn1c gene may induce a differentiation block at the DN3 to DN4 transition, and that the loss of this gene in the absence of *Tp53* led to aggressive thymic lymphomas.

**Results:**

In this manuscript, we demonstrated that the simultaneous deregulation of *CDKN1C*, *E2F1*, and *TP53* genes by epigenetic mechanisms and/or the deregulation of specific microRNAs, together with additional impairing of TP53 function by the expression of dominant-negative isoforms are common features in primary human T-LBLs.

**Conclusions:**

Previous experimental work in mice revealed that T-cell specific deletion of *Cdkn1c* accelerates lymphomagenesis in the absence of *Tp53*. If, as expected, the consequences of the deregulation of the CDKN1C-E2F1-TP53 axis were the same as those experimentally demonstrated in mouse models, the disruption of this axis might be useful to predict tumor aggressiveness, and to provide the basis towards the development of potential therapeutic strategiesin human T-LBL.

**Electronic supplementary material:**

The online version of this article (10.1186/s12885-018-4304-y) contains supplementary material, which is available to authorized users.

## Background

Precursor T-cell lymphoblastic neoplasms are aggressive haematological malignancies that mainly develop in children (in particular adolescent males) but also in adults. They derive from maturing thymocytes leading to excessive lymphoblastoid cells in the bone marrow and other lymphoid organs. Clinically, T-cell acute lymphoblastic leukaemia (T-ALL) and T-cell lymphoblastic lymphoma (T-LBL) are two subgroups differing by the extent of bone marrow infiltration. T-ALL manifests with extensive bone marrow and blood affectation, whereas a mass lesion in the thymus/anterior mediastinum with less than 25% of lymphoblasts in the bone marrow characterizes T-LBL [1].

As in other cancers, the loss of cell cycle control plays a prominent role in the pathogenesis of these malignancies that is primarily attributed to loss of *CDKN2A* (which encodes the tumour suppressor protein p16INK4A) and, to a lesser extent, loss of *RB1* or *CDKN1B* (which encodes p27/KIP1 protein) and aberrantly high levels of *CCND2* (encoding cyclin D2) [2]*.* Downregulation of *CDKN1C* (which encodes p57/KIP2 protein) by promoter hypermethylation has been detected with very low frequency in paediatric T-ALL and more often in adult patients. However, the biological and clinical impact of hypermethylation and/or loss of *CDKN1C* expression remain uncertain [3]. In addition to T-ALL, downregulation of *CDKN1C* has been observed more frequently in a wide variety of human tumours associated with a strengthening of cell proliferation [4, 5].

In addition, numerous studies have reported that *E2F1* overexpression has clinical relevance in many types of cancers [6]. However, to the best of our knowledge, *E2F1* alterations have not been so far implicated in the development of precursor T-cell neoplasms.

Moreover, the gene encoding TP53 protein, a main downstream effector of E2F1, is frequently targeted in human tumours by gene mutations [7, 8]. Apart from the canonical full-length transcript, it should be noted that alternative splicing of *TP53* and the use of alternate promoter might result in multiple transcript variants and isoforms [9] and, interestingly, abnormal expression of *TP53* isoforms has been reported in many cancers as head and neck, acute myeloid leukaemia (AML) and breast tumours [10] but not in T-cell lymphoblastic neoplasms.

The potential nexus between these three genes has been demonstrated in mice. Some authors [11] have shown in mouse models that inactivation of the *Cdkn1c* gene (also termed as *p57*^*KIP2*^) results in thymocyte development arrest at DN3 (Double-Negative 3) to DN4 cells transition, due to hyper-activation of the E2f-Tp53 pathway. Furthermore, the loss of *Cdkn1c* accelerates the development of thymic lymphomas in the absence of the *Tp53* gene.

To assess whether the axis *CDKN1C/E2F1/TP53* plays a role in human T-cell lymphoblastic lymphomas, we investigated the mutational status and the expression levels of these three genes using Next-Generation Sequencing (NSG) approaches. Interestingly, RNA-Sequencing analysis revealed reduced levels of *CDKN1C* mRNA in almost all analysed T-LBL samples, which may be accompanied by increased expression of *E2F1* and overexpression of the *TP53* transcript variant encoding the *∆133TP53* isoform. Deregulation of these genes is executed by epigenetics mechanisms and deregulation of specific miRNAs.

## Methods

### Human sample collection

Human T-LBL samples separated in an exploratory cohort (8 samples), an extended cohort (10 samples), and four thymuses of human foetus without haematological pathology, were obtained from the Spanish Hospital Biobanks Network (RetBioH; www.redbiobancos.es). Lymphomas were diagnosed according to World Health Organization Classification of Hematological Malignancies and recommendations from the European childhood lymphoma pathology panel [12, 13] (Additional file 1: Table S1). Institutional review board approval was obtained for these studies (reference CEI:70–1260).

### RNA-sequencing

Total RNA was obtained using TriPure Reagent (Roche Applied Science, Indianapolis, IN, USA), following manufacturer’s instructions.

#### Massive sequencing of mRNAs

RNA Integrity Numbers (RIN) were in the range of 7.2–9.8. Image analysis, per-cycle basecalling and quality score assignment were performed with Illumina Real Time Analysis software (Illumina, San Diego, CA). BCL files were converted to FASTQ format with Illumina’s Off-Line Basecaller package (Illumina). The resulting directional RNA-seq libraries were sequenced in paired-end format in two different rounds (Illumina HiSeq2000), leading to 50 bp and 76 bp reads (the latter were trimmed to 50 bp). Sequenced reads were quality-checked with FastQC (http://www.bioinformatics.babraham.ac.uk/projects/fastqc/). RNA-seq reads were aligned to the human genome (GRCh37/hg19) with TopHat-2.0.10 [14] (using Bowtie 1.0.0 [15] and Samtools 0.1.19 [16]) allowing two mismatches and five multihits. Transcripts assemblies, estimation of their abundances were calculated with Cufflinks 2.2.1, using the Ensembl GRCh37.74 annotation for human. In this analysis, we only considered the transcripts isoforms of the genes *CDKN1C*, *E2F1* and *TP53* that encode for proteins according to the information showed in Ensembl [17].

#### Small RNA

Image analysis and per-cycle basecalling was performed with Illumina Real Time Analysis software (RTA1.9) (Illumina). Conversion to FASTQ read format was performed by CASAVA-1.8 (Illumina). Small-RNA-seq libraries were sequenced as 40 bp single-end reads (Illumina Genome Analyzer IIx, GAIIx). Sequenced reads were quality-checked with FastQC. Sequence adapters were removed with cutadapt v1.2.1 [18] and only those reads longer than 15 bp and shorter than 35 bp were kept for further analysis. Reads were aligned to the human genome (GRCh37/hg19) with Bowtie 1.0.0 [15] and Samtools 0.1.19 [16] allowing no mismatches and a maximum of one alignment per read. Raw counts for miRNAs were obtained with HTSeq v0.5.3p9 [19], using the miRBase v20 [20] annotation for hg19. A table with normalized read counts was generated with DESeq [21] and was used to filter out miRNAs with questionable expression and outliers. The following criteria were used: first, we required that a miRNA should have a minimal normalized count value of 15 in at least 5% of the samples. Second, miRNAs with normalized expression values across the samples that exceeded Q1–3*IQR or Q3 + 3*IQR were considered outliers and discarded. For the remaining miRNAs, log2 fold-changes of expression were calculated.

Raw sequencing data and transcripts expression quantification is available as a superseries in GEO (Gene Expression Omnibus) under the following ID: GSE109234.

### Additional criteria to select miRNAs

To select those miRNA controlling *CDKN1C*, *E2F1* and *TP53* genes, we used the databases of miRGate and miRTarBase. We select those miRNAs experimentally validated (“Functional miRNA-target interactions (MTI)” registered in “Support type” of miRTarBase and/or “Functional MTI” registered in the miRGate “Confirmed predictions”) and/or those microRNA that showed a “miRGate Agreement Score” equal or higher than the median agreement-value of the microRNA identified associated with the genes assessed (median value = 1.04). (Additional file 2: Figure S1).

Additionally, we filtered out miRNAs showing a number of counts lower than 28.70 (median value of the miRNA counts of all the samples) in any sample (Additional file 3: Figure S2).

### Quantitative RT-PCR

RNA was reverse-transcribed using first the High-Capacity RNA-to-cDNA™ Kit (Applied Biosystems, Foster City, CA, USA) and MystiCq microRNA cDNA Synthesis Mix (Sigma-Aldrich, St. Louis, MO, USA). Quantitative real-time PCR reactions were performed in triplicate with an Applied Biosystems 7300 Real-Time PCR system (Life Technologies, Carlsbad, CA), using either the Fast Start Universal SYBRGreen Master (Rox) (Roche) or the MystiCq microRNA SYBR Green qPCR ReadyMix (Sigma-Aldrich), according to the manufacturers’ instructions. Expression values of β-2-microglobulin or β-actin or SNORD48 served to normalize using the 2-ΔΔC T method [22]. Primers are indicated in Additional file 4: Table S2.

### Targeted gene deep sequencing and sanger sequencing

Mutational status of *CDKN1C*, *E2F1* and *TP53* genes was analysed by targeted deep sequencing in genomic using a selected panel of cancer-related genes (the OncoNIM® Seq409 panel; New Integrated Medical genetics; NIMGenetics, Madrid, Spain). Sanger DNA sequencing of PCR-amplified mutational hot spots was performed with the specific primers summarized in Additional file 4: Table S2.

### Bisulfite genomic sequencing and methylation-specific PCR (MSP)

Methyl Primer Express v1.0 software (Applied Biosystems) was used to identify CpG islands around the Transcriptional Star Site (TSS) of *CDKN1C* gene, and to design specific primers for the methylation analysis. DNA (1 μg) was subjected to sodium bisulfite treatment using the EZ DNA Methylation-Gold kit (Zymo Research, CA, USA). MSP was performed with primers specific for methylated (M) or unmethylated (U) CpG sites. For bisulfite genomic sequencing, a region included in the one analysed by MSP was amplified using 1 μL of bisulfite-converted DNA with Immolase Taq polymerase (Bioline USA Inc., Kenilworth, NJ) at 60 °C for 40 cycles. Then the resulting PCR products were gel-purified (2% agarose) with Wizard® SV Gel and PCR Clean-Up System (Promega, Madison, WI, USA) and cloned into the pGEMT Easy Vector System (Promega) following the manufacturer-specific protocols. For all samples, 12 colonies were randomly chosen, and DNA was purified using Wizard® Plus SV Minipreps DNA Purification System (Promega) and sequenced with a ABI 3730 xl DNA Analyzer (Applied Biosystems). After sequencing analysis, the results were transformed into percentages of CpGs calculated in comparison with the total CpGs of the analysed region. Primers and conditions are indicated in Additional file 4: Table S2.

### Statistical analyses

Differential expression of mRNA and miRNA (RNA-Seq) between tumours and controls was estimated by calculating the log2 Fold changes (log2FC) of the expression levels. Only differential expression levels estimated by the Cufflinks software as “OK” were taken into account. Significant deregulated miRNAs with log2FC absolute values equal or higher than 1.5 in at least in one sample were selected according to the information of miRGate and miRTarBase databases [23, 24] and additional criteria based on the read counts [25]. Student’s t-test was used to compare results from qRT-PCR between tumours and controls. All statistical analyses were performed using R software.

## Results

### Deregulation *of CDKN1C*, *E2F1* and *TP53* in T-LBLs

**Fig. 1:**
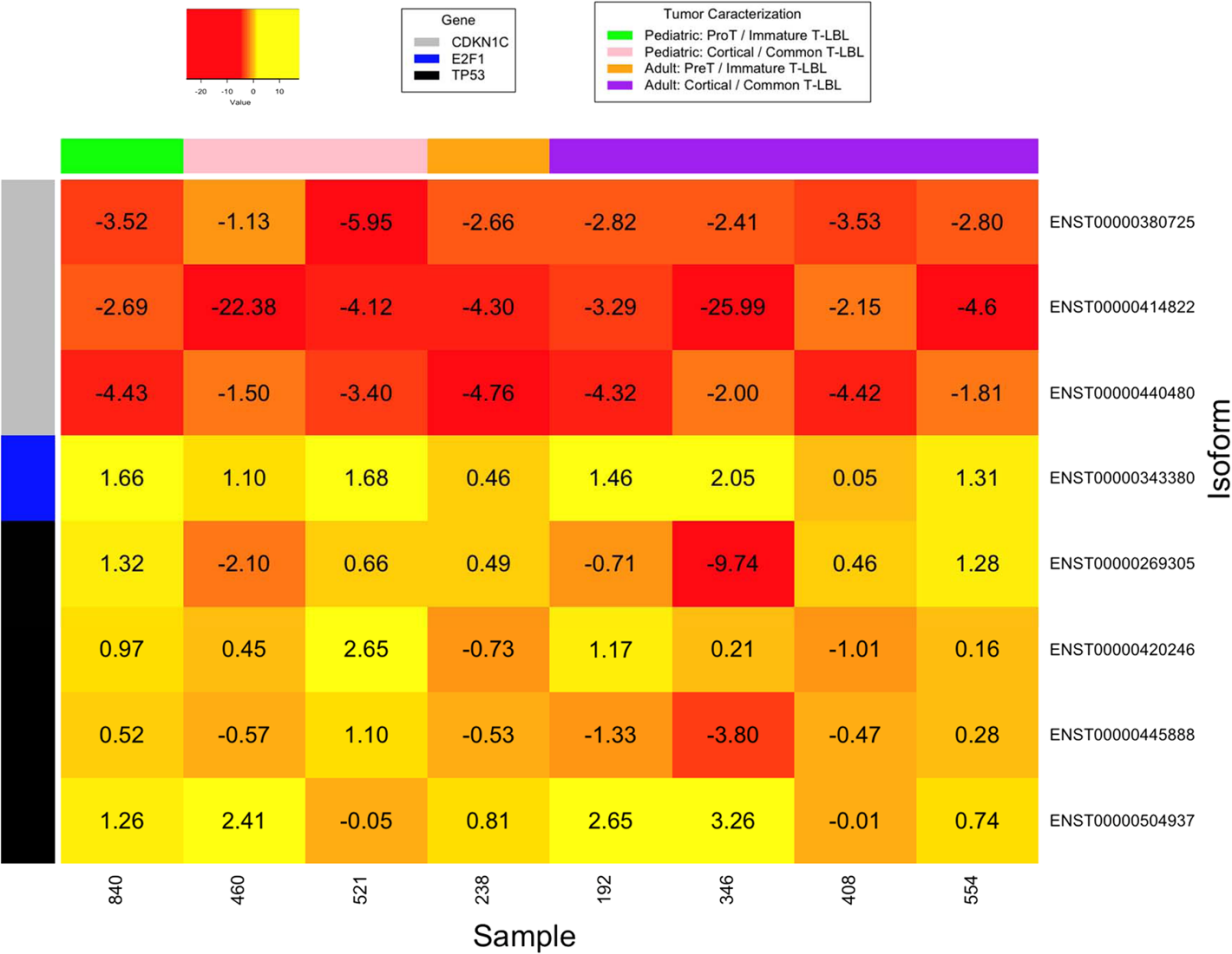
Deregulation of *CDKN1C*, *E2F1* and *TP53* in T-LBLs of the exploratory cohort by RNA-Seq. Numbers indicate log2 Fold changes (log2FC) between the expression of mRNAs in tumours and controls. Positive and negative values represent overexpression and reduced expression, respectively

**Fig. 2:**
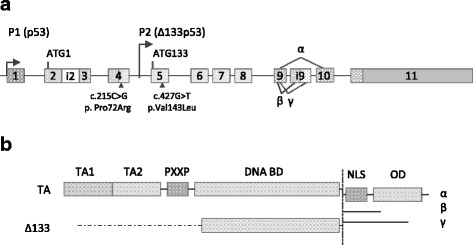
*TP53* mutations and isoforms showing differential expression in our sample series of T-LBL. **a** Genomic representation of the *TP53* gene showing the two missense mutations at exons 4 and 5 we detected in these samples. Alternative splicing of intron 9 generates p53 isoforms bearing different C-terminal domains (α, β and γ). **b** TP53 isoforms that showed differential levels of expression in our tumours. TAp53 isoforms include p53 (p53α), p53β and p53γ, whereas Δ133p53 isoforms include Δ133p53α, Δ133p53β and Δ133p53γ. TP53 protein domains: transactivation domains (TA), proline rich domain (PXXP), DNA binding domain (DNA BD), nuclear localization signal (NLS) and oligomerization domain (OD)

The results of massive RNA-sequencing (RNA-Seq) of the transcript isoforms that encode proteins in the 8 T-LBL samples of the exploratory cohort showed that the mRNA level of *CDKN1C* was strongly reduced in all analysed tumours compared to that of the normal foetal thymuses, with fold-changes ranging from − 25.99 to − 2.15 in the canonical isoform ENST00000414822. By contrary, the expression of *E2F1* gene in the same panel increases in all tumours, three of them with fold-changes higher than 1.5. Concerning the transcriptional status of the *TP53* gene, we found increased levels of the transcript variant encoding the dominant-negative ∆133p53α protein isoform (TP53–008: ENST00000504937) in all but two of the eight tumours (fold-changes between 3.26 and 0.74). Moreover, two of them (346 and 460) exhibited a clear reduction of two transcript variants (TP53–001: ENST00000269305 and TP53–002: ENST00000445888) encoding full-length TP53 protein isoforms (TAp53α). Finally, two tumours (192 and 521) showed increased amounts of the TP53β transcript (ENST00000420246), which encodes a C-terminal truncated protein (Figs. 1 and 2; Additional file 5: Table S3).

**Fig. 3:**
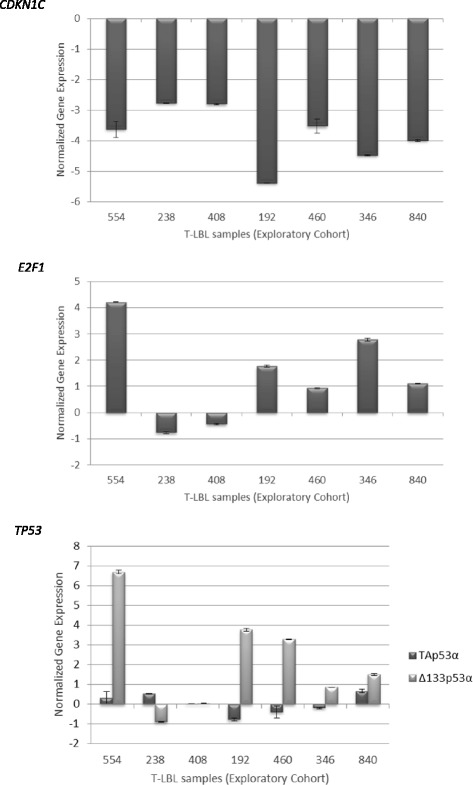
Differential expression of *CDKN1C, E2F1* and *TP53* in T-LBLs of the exploratory cohort by quantitative RT–PCR. Relative expression values were calculated as the mRNA amount of each gene relative to that of either β-actin or β2 microglobulin (used as reference) and normalized to the relative expression of normal control samples (foetal thymuses). Each bar represents the mean ± SD of three independent experiments. Differences in expression values were statistically significant (*p* < 0.05)

These results were validated by quantitative real-time RT-PCR (qRT-PCR) analysis and confirmed in the extended cohort (Fig. 3; Additional file 6: Table S4). Interestingly, all samples in the extended cohort showed a significant reduction of *CDKN1C* expression, six out of ten showed significant increases of *E2F1* expression, and six out of ten exhibited significant increases of the mRNA isoform encoding ∆133p53α protein.

### Overrepresentation of the arginine allele at codon 72 of *TP53* in T-LBLs

The analysis of T-LBLs by targeted gene deep sequencing revealed the existence of two missense mutations. One of them was c.427G > T (p.Val143Leu) at exon 5 in sample 192, with conflicting interpretations of pathogenicity in the IARC database [26]. The other missense mutation was the functional polymorphism c.215C > G (p.Pro72Arg) that was found in all but one analysed tumours (8/9), three of them being homozygotes for the arginine allele (238, 521, and 840) (Additional file 7: Table S5) (Fig. 2). However, we were able to validate only the mutation at exon 4 by DNA Sanger-sequencing (data not shown) using the primers and conditions indicated in Additional file 4: Table S2.

### Epigenetic modifications contribute to the altered expression of *CDKN1C* in a fraction of T-LBLs

**Fig. 4:**
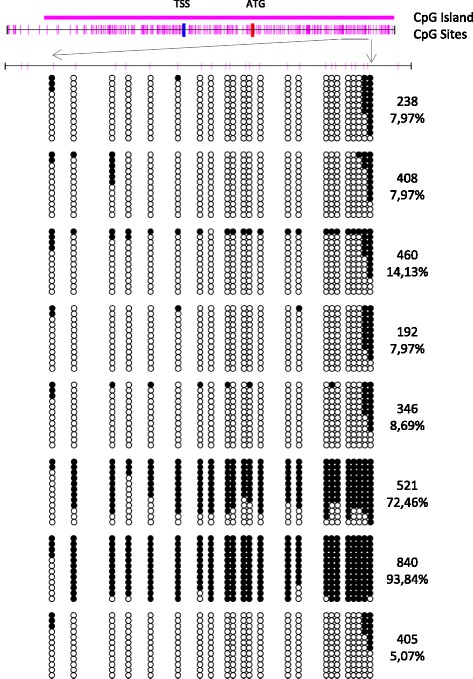
Schematic depiction of the CpG-island around the transcription start site of *CDKN1C* (TSS). ATG indicate the position of the translation start site. Short vertical lines represent CpG dinucleotides. Methylated (black circles) or unmethylated (white circles) CpG sites are indicated in 12 sequenced clones for every tumour. Methylation density is indicated as the percentage of methylated sites in comparison with total CpG sites

The *CDKN1C* gene is remarkably rich in CpG islands situated both upstream and downstream from the transcriptional start site, whose hypermethylation has been strongly related to its inactivation [27]. To elucidate whether aberrant DNA methylation is a mechanism whereby *CDKN1C* was downregulated in our T-LBLs, we analysed the DNA methylation levels of the promoter region of *CDKN1C* gene using the MSP/Sequencing method. We initially examined six samples of normal foetal thymuses by MSP, confirming the absence of methylated bands in all cases (data not shown). However, although all samples in the exploratory cohort exhibited significant downregulation of this gene, only two tumours exhibited high levels of hypermethylation (521 and 840), suggesting additional mechanisms to explain *CDKN1C* downregulation in the remaining samples (Fig. 4).

### MicroRNA deregulation contributes to the deregulation of *CDKN1C*, *E2F1* and *TP53* genes in T-TLBLs

Aberrant expression of microRNAs (miRNAs) in human tumours and links between deregulated miRNAs and target genes involved in cell cycle have been well established [28, 29].

**Fig. 5:**
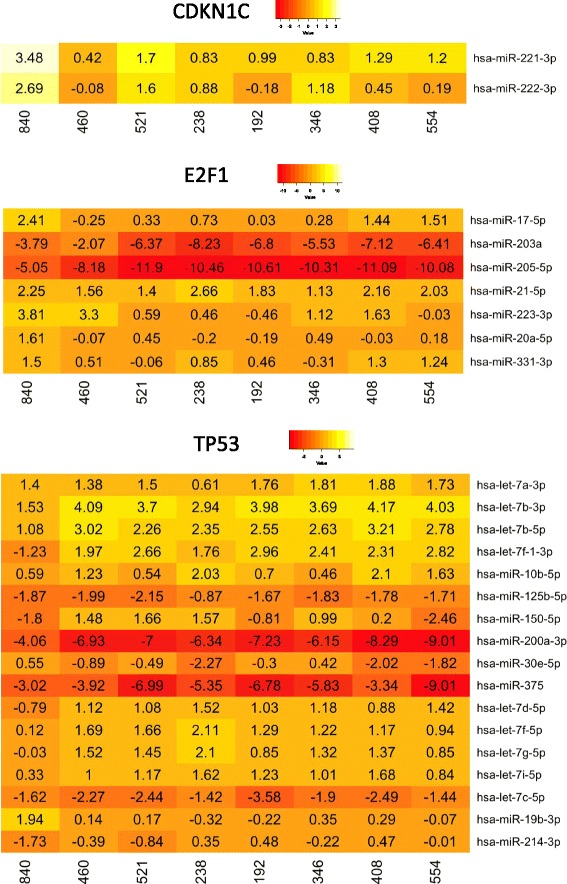
Deregulated MicroRNA controlling *CDKN1C*, *E2F1* and *TP53* genes in T-LBLs of the exploratory cohort (RNA-Seq). Numbers indicate log2 Fold changes (log2FC) between the miRNAs read counts in tumours and controls. Positive and negative values represent up-regulated and down-regulated, respectively. Only miRNAs showing log2FC absolute values equal or higher than 1.5 in at least in one sample are depicted. The number in each column represents the sample identifier

Differences in the expression level of the selected miRNA were initially determined by massive small RNA-sequencing (RNA-Seq) in the 8 T-LBL samples of the exploratory cohort. There were only two significant deregulated miRNAs controlling *CDKN1C* expression*,* which are up-regulated in practically all the samples (miR-221–3p and miR-222-3p). We also found seven deregulated miRNAs to target *E2F1* transcript, especially emphasizing the importance of miR-203a and miR-205-5p that were strongly down-regulated. Finally, 17 miRNAs to target *TP53* transcripts were deregulated highlighting the levels of miR-200a-3p and miR-375 downregulation of (Fig. 5; Additional file 8: Table S6).

**Fig. 6:**
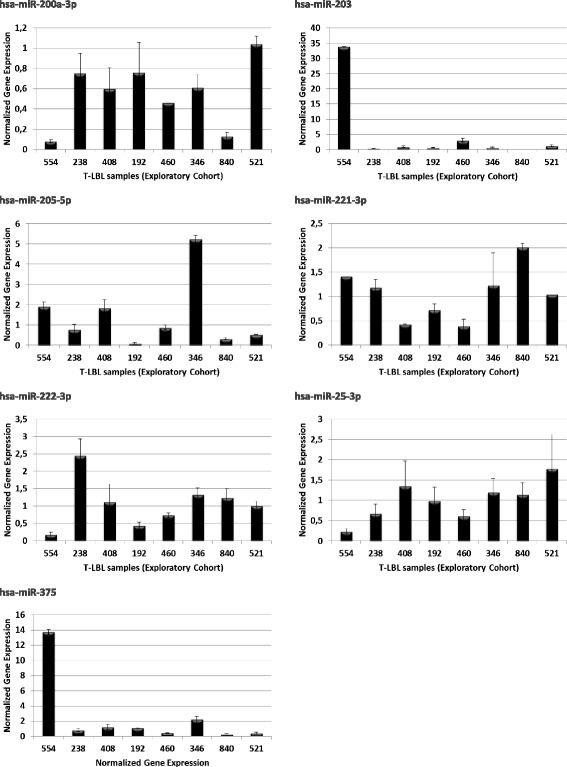
Differential expression of miRNAs regulating *CDKN1C, E2F1* and *TP53* expression by interaction with its 3’UTR. Transcriptional levels of hsa-miR-200a-3p, hsa-miR-203, hsa-miR-205-5p, hsa-miR-221–3p, hsa-miR-222-3p, hsa-miR-25-3p and in human T-LBLs were measured using qRT-PCR assay. Relative expression values were calculated as the mRNA amount of each gene relative to miR-SNORD48 (used as reference) and normalized to the relative expression of normal control samples (foetal thymuses). Each bar represents the mean ± SD of three independent experiments. Differences in expression values were statistically significant (*p* < 0.05)

Deregulation of these miRNAs was confirmed by qRT-PCR in the exploratory cohort and in the extended cohort of T-LBL samples (Fig. 6; Additional file 9: Table S7).

## Discussion

It is well established that *CDKN1C* and *E2F1* are two critical controllers of the cell cycle. The overexpression of *CDKN1C* may cause cell cycle arrest in human tumour cell lines [30, 31], and this inhibitory effect may be reversed by siRNAs against the *CDKN1C* gene [32]. In contrast, knockdown of *E2F1* by RNA interference impairs proliferation of rat glioma cells [33]. Importantly, previous experimental work in mice reported that conditional T cell-specific deletion of *Cdkn1c* gene induced a differentiation block in mouse immature thymocytes that is caused by hyperactivation of *E2f1* and *Tp53* and may be predisposed to thymic lymphoma development. Moreover, *Cdkn1c* ablation led to the development of aggressive thymic lymphomas with a reduced latency in a *Tp53*-null background. Thus, these results suggested a critical role for the *Cdkn1c-E2f1-Tp53* axis in mouse thymic lymphoma development [11, 34].

Our results show that all analysed human T-LBL samples exhibited a strong downregulation of *CDKN1C*. In addition, most of them also exhibited upregulation of *E2F1* (6/8 in the exploratory cohort and 6/10 in the extended cohort), which may be accompanied by impairment of TP53 function in some cases (4/6 in the exploratory cohort and 6/10 in the extended cohort) (Fig. 1; Additional file 3: Table S3 and Additional file 4: Table S4)*.* Thus, our data are consistent with the existence and deregulation of a *CDKN1C-E2F1-TP53* axis in human T-LBL. However, it should be noted that our study is largely based on the expression of these genes at the transcriptional level. The relationship between mRNA and protein expression levels is dependent on the combined outcomes of mRNA stability, translation, and protein degradation. Notwithstanding, it has been reported that at least 30 to even 85% of the variation in protein levels can be attributed to variation in mRNA expression [35]. Other authors [36] reported that differentially expressed mRNAs correlate significantly better with their protein product than non-differentially expressed mRNAs, therefore providing some optimism for the usefulness on inferences from mRNA expression in general.

Concerning the mechanisms by which these genes are deregulated, it is well known that *CDKN1C* is subject to a complex regulation involving the cooperation of a CpG island at its promoter region and distal regulatory elements, such as the imprinting control region Kv-Differentially Methylated Region 1 (KvDMR1) in the promoter of the noncoding *KCNQ1OT1* [37, 38]. Although the biological and clinical impact of *CDKN1C* hypermethylation is rather uncertain, aberrant DNA methylation of *CDKN1C* in its promoter region has been reported in lymphoid malignancies of B and T-cell phenotype [39, 40]. However, *CDKN1C* has been reported downregulated in other type of cancer cells mainly by histone modifications operating in critical regions of its promoter [41, 42]. We initially focused on promoter hypermethylation to explain downregulation of this gene in our sample series of T-LBL, but despite a substantial reduction in the levels of mRNA in almost all samples in the exploratory cohort (7/8), only two samples (840 and 521) (2/8) exhibited significant hypermethylation density (Fig. 4), and six out of eight (including tumor 840 with promoter hypermethylation) exhibited upregulation of one or two miRNAs selected for *CDKN1C* regulation (miR-211–3p and miR-222-3p). Thus, downregulation of *CDKN1C* in two samples (33 and 346) should be explained by a different transcriptional mechanism.

Besides this epigenetic mechanism, regulation by miRNAs might be an additional way contributing to determine *CDKN1C* transcript levels in T-LBLs. Results reported here are in line with those reported in the literature describing miR-25, miR-221 and miR-222 as direct regulators of *CDKN1C* expression in a wide variety of solid tumours, showing a new mechanism responsible for *CDKN1*C downregulation in carcinogenesis [43–45]. In this context, our findings suggest that aberrant expression of miR-221 and miR-222 may have an oncogenic function in T-LBL development by targeting *CDKN1C*. However two samples (33 and 346) showed a pronounced downregulation of *CDKN1C* in the absence of significant changes in miRNA expression (Figs. 5 and 6) or promoter CpG methylation, thus indicating that the mechanism regulating the expression of this gene is far more complex.

Overexpression of *E2F1* may promote proliferation or cell cycle progression by increasing the transcription of genes that contribute to G1-S transition [46]. Notwithstanding at the same time it may also induce apoptosis by multiple pathways, some of which induce stabilization and activation of the TP53 protein [47]. Our microRNA analysis also revealed a consistent deregulation of seven miRNAs in T-LBLs, miR-203a and miR-205-5p being the most representative downregulated microRNAs (Figs. 5 and 6). Interestingly, downregulated miRNAs showed higher fold changes than upregulated microRNAs. miR-205-5p is known to be down-regulated in melanoma and its expression inversely correlated with that of *E2F1* [48].

Concerning impairing of *TP53* function, we found overexpression of the human *Δ133p53α*isoform in 4 samples from the exploratory cohort, from which three also exhibited downregulation of the isoform encoding full length TAp53α protein isoforms (Figs. 1 and 2). It has been demonstrated that *∆133p53α* does not exclusively function in a dominant-negative manner toward *TAp53α*, the full-length *TP53* isoform [49], but it also inhibits *TP53*-dependent apoptosis [50]*.* Finally, two tumours (192 and 521) showed increased amounts of the *TP53β* transcript, which encodes a C-terminal truncated protein that downplay TP53 capacity to induce apoptosis [9, 51]. These changes in the expression levels of full length and shorter isoforms may be sustained, at least in part, by deregulation of 17 miRNAs, with particular reference to miR-200a-3p and miR-375 that exhibited very high levels of downregulation in all samples in the exploratory cohort (Figs. 5 and 6).

But impairment of the *TP53* function could be also attributed to the overrepresentation of the arg72 allele in our sample series (Fig. 2)*.* It is known that the *TP53* gene is not only frequently mutated in human tumours [7], but it also contains several functional polymorphisms, being by far the most common a proline (Pro) to arginine (Arg) change at codon 72 in the TP53 protein [10]. Several studies have reported preferential retention of arg72 allele in squamous cell carcinomas of the vulva [52], head and neck [53], and esophagus [54]. Considering tumour tissue DNA, Schneider-Stock et al. [55] found a significantly higher frequency of the arg72 allele in colorectal tumours and reported that the presence of this allele correlates with the malignant potential of the tumour. Similar results were also reported in urinary tract cancers [56] and lung cancer [57]. The arg72 allele was also related with increased risk for bladder cancer [58].

## Conclusions

**Fig. 7:**
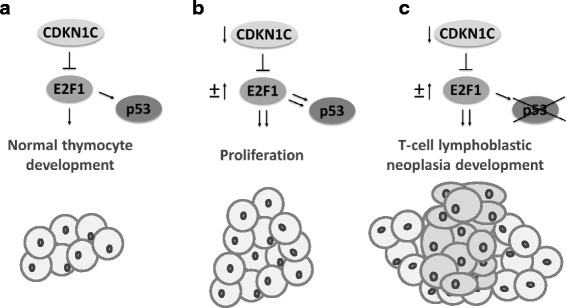
*CDKN1C-E2F1-TP53* axis in T-LBL development. Having in mind the consequences of alterations in these genes in mouse models [11, 34], T-LBLs in our samples series might be classified into different categories. **a** In normal cells *CDKN1C* regulates *E2F1* to control the expression of *E2F* target genes and the activity of TP53 during thymocyte development. **b** Decreasing of *CDKN1C* (due to epigenetic mechanisms and/or upregulation of specific miRNAs) could lead to proliferation, that may be favoured by overexpression of E2F1 (through downregulation of specific miRNAs). **c** The additional impairment of TP53 (by the combined effect of inactivating mutations, differential expression of isoforms, and/or deregulation of specific miRNAs) should lead T-cell lymphoblastic neoplasia development

Our results indicate the existence of a *CDKN1C*-*E2F1*-*TP53* axis that is disrupted in a significant fraction of human T-LBLs. If, as expected, the consequences of the deregulation of the CDKN1C-E2F1-TP53 axis were the same as those experimentally demonstrated in mouse models, deregulation of this axis in human T-LBL might serve as a biomarker to predict the aggressiveness of T-LBL development as depicted in Fig. 7. Furthermore, these findings would provide the basis to the development of potential therapeutic strategies based on the use of microRNAs (mimics or antagomirs) to target *CDKN1C* or *E2F1* deregulation that allow to rescue normal thymocyte differentiation and normal levels of thymocytes proliferation in patients with T-LBL. Blocking *E2F1* expression by RNA interference might represent a promising therapeutic approach in this type of tumours. Future studies with new samples series of T-LBL should be done to be sure that the differences detected at the mRNA level translate into the protein level, and to confirm that the deregulation of this axis in human samples is really predictive of clinical outcome.

## Additional files

Additional file 1: **Table S1.** Characterization of the human sample collection in the exploratory cohort. Lymphomas were diagnosed (see Characterization column) according to World Health Organization Classification of Hematological Malignancies and recommendations from the European. (PDF 205 kb)

Additional file 2: **Figure S1.** Gaussian Kernel Density plot of miRGate Agreement Score of the miRNAs identified associated with the *CDKN1C*, *E2F1* and *TP53* genes. (PDF 283 kb)

Additional file 3: **Figure S2.** Gaussian Kernel Density Plot of the red counts for the miRNAs deregulated in any sample. (PDF 1208 kb)

Additional file 4: **Table S2.** Description of primers used in qRT-PCR, Targeted gene deep sequencing, Sanger sequencing and Methylation-Specific PCR. (PDF 95 kb)

Additional file 5: **Table S3.** Differential expression of mRNA between tumours and controls in the exploratory cohort by RNA-Seq. (PDF 77 kb)

Additional file 6: **Table S4.** Relative expression of CDKN1C, E2F1 and TP53 analyzed by qrtRT-PCR. (PDF 88 kb)

Additional file 7: **Table S5.** Complete list of genetic variants for TP53 gene determined by targeted deep sequencing in the T-LBL samples. (PDF 96 kb)

Additional file 8: **Table S6.** MicroRNA regulation of CDKN1C, E2F1 and TP53 genes in T-TLBLs of the exploratory cohort by RNA-Seq. (PDF 78 kb)

Additional file 9: **Table S7.** Validation analysis by qRT-PCR of those miRNA significantly deregulated according with RNA-Seq analysis in the exploratory and extended cohort of T-LBL samples. (PDF 81 kb)

## Abbreviations

AML: Acute myeloid leukaemia
DN3: Double-Negative 3
DN4: Double-Negative 4
FC: Fold-change
FDR: False discovery rate
GEO: Gene Expression Omnibus
KvDMR1: Kv-Differentially Methylated Region 1
miRNAs: microRNAs
MTI: miRNA-target interactions
NSG: Next-Generation Sequencing
qRT-PCR: Quantitative reverse transcription polymerase chain reaction),
RIN: RNA Integrity Numbers
RNA-Seq: Massive RNA-sequencing
RT-PCR: Reverse transcription polymerase chain reaction
T-ALL: T-cell acute lymphoblastic leukaemia
T-LBL: T-cell lymphoblastic lymphomas
