# Supplementary material for: RNA-Seq reveals the existence of a CDKN1C-E2F1-TP53 axis that is altered in human T-cell lymphoblastic lymphomas
Source: BMC Cancer. 2018 Apr 16;18:430. doi: 10.1186/s12885-018-4304-y (PMC5902834; doi:10.1186/s12885-018-4304-y)
Supplement: Supplementary file 1 — Table S1. Characterization of the human sample collection in the exploratory cohort. Lymphomas were diagnosed (see Characterization column) according to World Health Organization Classification of Hematological Malignancies and recommendations from the European. (PDF 205 kb) [file 12885_2018_4304_MOESM1_ESM.pdf]

Supplementary Table 1. Characterization of the human sample collection in the exploratory cohort. Lymphomas were diagnosed (see Characterization column) according to World Health Organization Classification of Hematological Malignancies and recommendations from the European

								**CDKN1C**	**CDKN1C**	**CDKN1C**	**E2F1**	**TP53**	**TP53**	**TP53**	**TP53**
					**qrtRT-PCR**			**ENST00000380725**	**ENST00000414822**	**ENST00000440480**	**ENST00000343380**	**ENST00000269305**	**ENST00000420246**	**ENST00000445888**	**ENST00000504937 (Δ)**
	**IDENTIFICATION CODE***	**Age**	**Tumor cells**	**Characterization**	**CDKN1C**	**E2F1**	**TP53**	**CDKN1C-002**	**CDKN1C-001**	**CDKN1C-003**	**E2F1-001**	**TP53-001**	**TP53-005**	**TP53-002**	**TP53-008**
Exploratory cohort	554	Adult	>80%	Cortical / Common T-LBL	-3,625	4,214	0,31	-2,80	-4,60	-1,81	1,31	1,28	0,16	0,28	0,74
	238	Adult	>80%	PreT / Immature T-LBL	-2,766	-0,761	0,536	-2,66	-4,30	-4,76	0,46	0,49	-0,73	-0,53	0,81
	408	Adult	>80%	Cortical / Common T-LBL	-2,795	-0,434	0,028	-3,53	-2,15	-4,42	0,05	0,46	-1,01	-0,47	-0,01
	192	Adult	90%	Cortical / Common T-LBL	-5,38	1,769	-0,78	-2,82	-3,29	-4,32	1,46	-0,71	1,17	-1,33	2,65
	346	Adult	95%	Cortical / Common T-LBL	-4,473	2,78	-0,184	-2,41	-25,99	-2,00	2,05	-9,74	0,21	-3,80	3,26
	460	Pediatric	70%	Cortical / Common T-LBL	-3,522	0,933	-0,395	-1,13	-22,38	-1,50	1,10	-2,10	0,45	-0,57	2,41
	840	Pediatric	>80%	ProT / Immature T-LBL	-3,988	1,104	0,659	-3,52	-2,69	-4,43	1,66	1,32	0,97	0,52	1,26
	521	Pediatric	90%	Cortical / Common T-LBL				-5,95	-4,12	-3,40	1,68	0,66	2,65	1,10	-0,05
Extended cohort	829	Adult	—	Immature T-LBL	-3,023	-0,436	0,171								
	188	Adult	—	T-LBL	-3,251	1,046	0,046								
	135	Adult	—	Medular / Common T-LBL	-5,107	1,742	-0,295								
	080	Pediatric	—	Medular / Common T-LBL	-3,21	0,029	-0,666								
	104	Pediatric	—	Cortical / Common T-LBL	-4,975	1,015	1,773								
	038	Pediatric	—	T-LBL	-0,682	1,257	0,522								
	101	Pediatric	—	T-LBL	-3,736	1,704	-1,689								
	209	Pediatric	—	T-LBL	-1,187	0,453	0,389								
	639	Pediatric	80%	Medular / Common T-LBL	-2,498	-0,479	0,07								
	001	Pediatric	—	ProT-PreT / Immature T-LBL	-2,766	2,651	0,547								

*Identification of the biological samples of the Biobank will be subjected to a coding process. Each sample is assigned an identification code.
