# Supplementary material for: RNA-Seq reveals the existence of a CDKN1C-E2F1-TP53 axis that is altered in human T-cell lymphoblastic lymphomas
Source: BMC Cancer. 2018 Apr 16;18:430. doi: 10.1186/s12885-018-4304-y (PMC5902834; doi:10.1186/s12885-018-4304-y)

Fig S1. Gaussian Kernel Density plot of miRGate Agreement Score of the miRNAs identified associated with the *CDKN1C*, *E2F1* and *TP53* genes. Red dashed line represents the median value (1.04) of the Agreement Scores. A= Gaussian Kernel Density plot of miRGate Agreement Score of all the microRNAs; B= Gaussian Kernel Density plot of miRGate Agreement Score only for the microRNAs experimentally validated according with the information shown in miRGate..

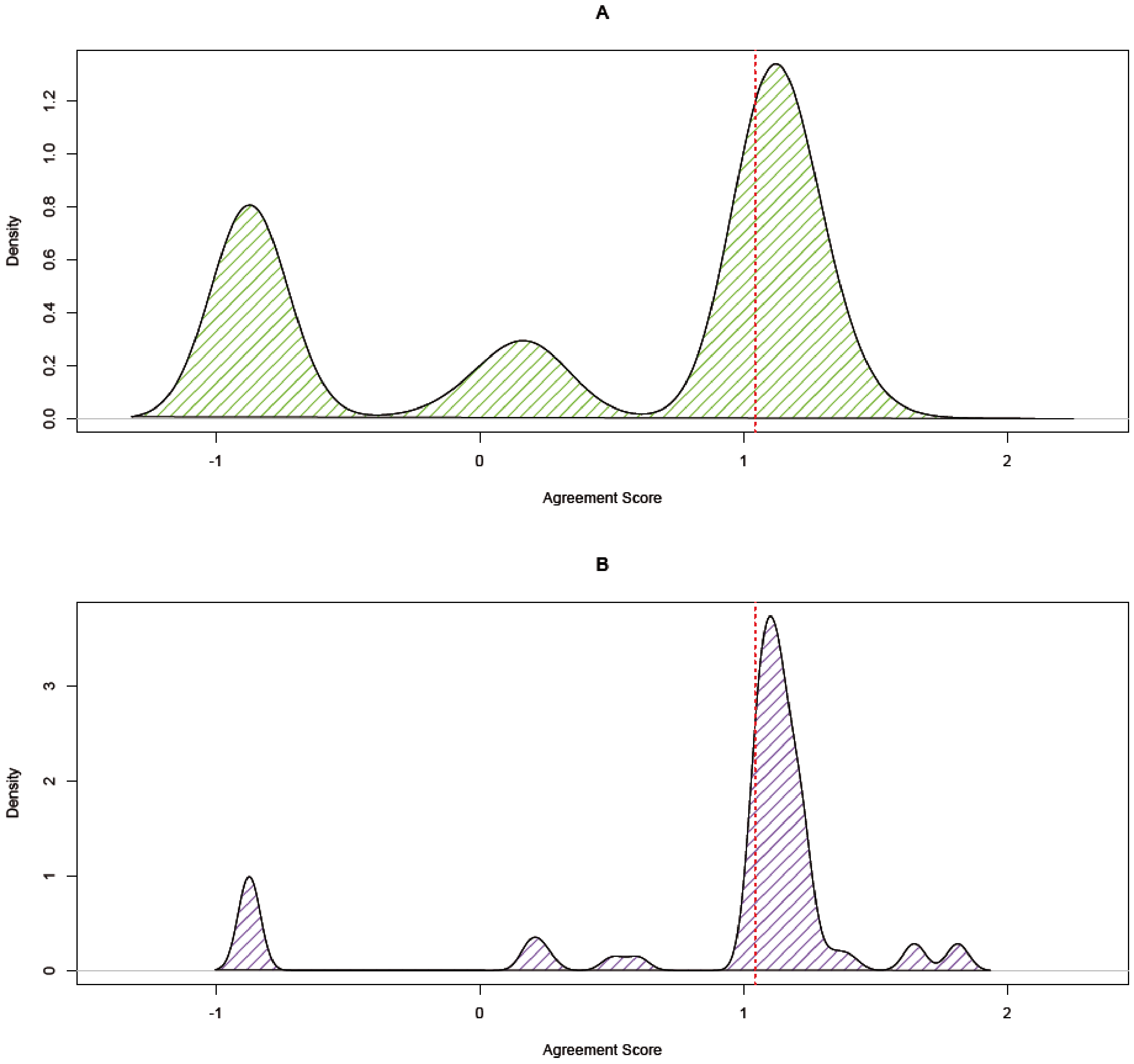

Supplement: Supplementary file 2 — Figure S1. Gaussian Kernel Density plot of miRGate Agreement Score of the miRNAs identified associated with the CDKN1C, E2F1 and TP53 genes. (PDF 283 kb) [file 12885_2018_4304_MOESM2_ESM.pdf]
