# Supplementary material for: RNA-Seq reveals the existence of a CDKN1C-E2F1-TP53 axis that is altered in human T-cell lymphoblastic lymphomas
Source: BMC Cancer. 2018 Apr 16;18:430. doi: 10.1186/s12885-018-4304-y (PMC5902834; doi:10.1186/s12885-018-4304-y)

Fig S2. Gaussian Kernel Density Plot of the read counts for the miRNAs deregulated in any sample. Black dashed line represents the median value (28.70) of the miRNA read counts of all the samples and red line represents the mean value of the controls read counts.

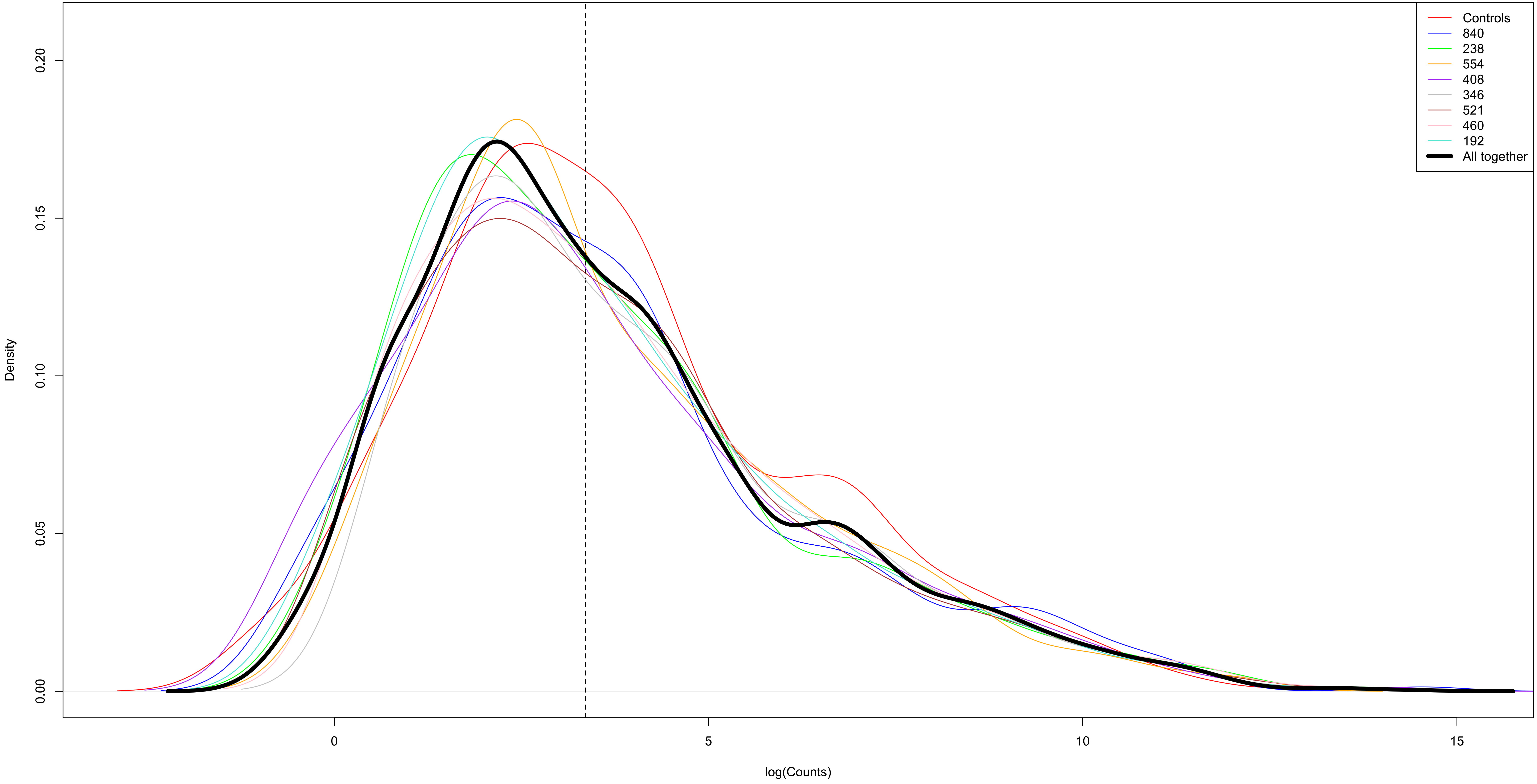

Supplement: Supplementary file 3 — Figure S2. Gaussian Kernel Density Plot of the red counts for the miRNAs deregulated in any sample. (PDF 1208 kb) [file 12885_2018_4304_MOESM3_ESM.pdf]
