# Supplementary material for: RNA-Seq reveals the existence of a CDKN1C-E2F1-TP53 axis that is altered in human T-cell lymphoblastic lymphomas
Source: BMC Cancer. 2018 Apr 16;18:430. doi: 10.1186/s12885-018-4304-y (PMC5902834; doi:10.1186/s12885-018-4304-y)
Supplement: Supplementary file 4 — Table S2. Description of primers used in qRT-PCR, Targeted gene deep sequencing, Sanger sequencing and Methylation-Specific PCR. (PDF 95 kb) [file 12885_2018_4304_MOESM4_ESM.pdf]

Supplementary Table 2. Description of primers used in qRT-PCR, Targeted gene deep sequencing, Sanger sequencing and Methylation-Specific PCR.

Primer sequences qRT-PCR

Target gene	Primer sequences (5'-3')	Size
E2F1[1]	CATCCCAGGAGGTCACTTCTG	144
	GACAACAGCGGTTCTTGCTC	
P57[2]	GGCCTCTGATCTCCGATTTCTTC	126
	GGGTCTGCTCCACCGAG	
*T*A*p53*[3]	CGCAGTCAGATCCTAGCGTC	171
	CTGGACCTGGGTCTTCAGTG	
Δ133*p* 53[4]	GGTTGCAGGAGGTGCTTACAC	128
	GTTGAGGGCAGGGGAGTACTG	
β2μG	CCAGCAGAGAATGGAAAGTC	285
	CGAGACATGTAAGCAGCATC	
β actin	CAGGCACCAGGGCGTG	84
	GTGAGGATGCCTCTCTTGCTCT	

MystiCQ™ microRNA qPCR

	Target	SIGMA Reference
	Universal PCR Primer	MIRUP
	Assay Primer hsa-miR-25-3p	MIRAP00059
	Assay Primer hsa-miR-200a-3p	MIRAP00247
	Assay Primer hsa-miR-203	MIRAP00255
	Assay Primer hsa-miR-205-5p	MIRAP00257
	Assay Primer hsa-miR-221-3p	MIRAP00277
	Assay Primer hsa-miR-222-3p	MIRAP00279
	Assay Primer hsa-miR-375	MIRAP00360
	Control Primer SNORD48	MIRCP00007

Primer sequences for Sanger DNA sequencing

Target Gene	Region	Sequence 5'-3'	Size
TP53	Exon 4[5]	ACTGCTCTTTTCACCCATCTAC	332
		TCATGGAAGCCAGCCCCTCAG	
	Exon 5	GGTGTAGACGCCAACTCTCTC	340
		CCAGCTGCTCACCATCGCTA	
	Exon 6[6, 7]	TGGTTGCCCAGGGTCCCC	223
		CGGAGGGCCACTGACAACC	
	Exon 7[8]	TGCCACAGGTCTCCCCAAGG	196
		AGTGTGCAGGGTGGCAAGTG	
	Exon 8	GCTTTGAGGTGCGTGTTTGT	170
		CTGAGGCATAACTGCACCCT	

* European Molecular Biology Laboratory (EMBL) Accession No. X54156.1 (p53 gene sequence).

Primer sequences for Methylation-Specific PCR and Bisulfite Genomic Sequencing

Target Gene		Sequence 5'-3'	Size
P57	MSP-U-F	GTTGAATGTTGAGGATTAGAATTGT	203
	MSP-U-R	CAAAAAACAAACTAACAACCACCAC	
	MSP-M-F	AGTTGAACGTCGAGGATTAGAATC	203
	MSP-M-R	GAAAAACGAACTAACAACCACCG	
	BS-F	GAGGATTAGAATYGTTGGGATT	184
	BS-R	AACTAACAACCACCRCRA	

References:

1. Wang P, Alvarez-Perez JC, Felsenfeld DP, et al. A high-throughput chemical screen reveals that harmine-mediated inhibition of DYRK1A increases human pancreatic beta cell replication. Nat Med. 2015;21(4):383-388.
2. Ueberberg S, Tannapfel A, Schenker P, et al. Differential expression of cell-cycle regulators in human beta-cells derived from insulinoma tissue. Metabolism. 2016;65(5):736-746.
3. Nutthasirikul N, Limpaiboon T, Leelayuwat C, Patrakitkomjorn S, Jearanaikoon P. Ratio disruption of the 133p53 and TAp53 isoform equilibrium correlates with poor clinical outcome in intrahepatic cholangiocarcinoma. Int J Oncol. 2013;42(4):1181-1188.
4. Ohgaki H, Dessen P, Jourde B, et al. Genetic pathways to glioblastoma: a population-based study. Cancer Res. 2004;64(19):6892-6899.
5. Bharaj BS, Angelopoulou K, Diamandis EP. Rapid sequencing of the p53 gene with a new automated DNA sequencer. Clin Chem. 1998;44(7):1397-1403.
6. Serakinci N, Christensen R, Graakjaer J, et al. Ectopically hTERT expressing adult human mesenchymal stem cells are less radiosensitive than their telomerase negative counterpart. Exp Cell Res. 2007;313(5):1056-1067.
7. Millikan R, Hulka B, Thor A, et al. p53 mutations in benign breast tissue. J Clin Oncol. 1995;13(9):2293-2300.
