# Supplementary material for: RNA-Seq reveals the existence of a CDKN1C-E2F1-TP53 axis that is altered in human T-cell lymphoblastic lymphomas
Source: BMC Cancer. 2018 Apr 16;18:430. doi: 10.1186/s12885-018-4304-y (PMC5902834; doi:10.1186/s12885-018-4304-y)
Supplement: Supplementary file 5 — Table S3. Differential expression of mRNA between tumours and controls in the exploratory cohort by RNA-Seq. (PDF 77 kb) [file 12885_2018_4304_MOESM5_ESM.pdf]

Supplementary Table 3. Differential expression of mRNA between tumours and controls in the exploratory cohort by RNA-Seq.

Gene Symbol	Ensembl_gene_ID	Ensembl_transcript_ID	Locus	Tumor	FPKM_tumor	FPKM_control	log2FC
CDKN1C	ENSG00000129757	ENST00000380725	chr11:2904442-2907111	840	0,764	8,779	-3,52
CDKN1C	ENSG00000129757	ENST00000380725	chr11:2904442-2907111	192	1,240	8,779	-2,82
CDKN1C	ENSG00000129757	ENST00000380725	chr11:2904442-2907111	460	4,009	8,779	-1,13
CDKN1C	ENSG00000129757	ENST00000380725	chr11:2904442-2907111	346	1,653	8,779	-2,41
CDKN1C	ENSG00000129757	ENST00000380725	chr11:2904442-2907111	521	0,142	8,779	-5,95
CDKN1C	ENSG00000129757	ENST00000380725	chr11:2904442-2907111	238	1,390	8,779	-2,66
CDKN1C	ENSG00000129757	ENST00000380725	chr11:2904442-2907111	554	1,265	8,779	-2,80
CDKN1C	ENSG00000129757	ENST00000380725	chr11:2904442-2907111	408	0,758	8,779	-3,53
CDKN1C	ENSG00000129757	ENST00000414822	chr11:2904442-2907111	840	0,399	2,577	-2,69
CDKN1C	ENSG00000129757	ENST00000414822	chr11:2904442-2907111	192	0,264	2,577	-3,29
CDKN1C	ENSG00000129757	ENST00000414822	chr11:2904442-2907111	460	0,000	2,577	-22,38
CDKN1C	ENSG00000129757	ENST00000414822	chr11:2904442-2907111	346	0,000	2,577	-25,99
CDKN1C	ENSG00000129757	ENST00000414822	chr11:2904442-2907111	521	0,148	2,577	-4,12
CDKN1C	ENSG00000129757	ENST00000414822	chr11:2904442-2907111	238	0,131	2,577	-4,30
CDKN1C	ENSG00000129757	ENST00000414822	chr11:2904442-2907111	554	0,106	2,577	-4,60
CDKN1C	ENSG00000129757	ENST00000414822	chr11:2904442-2907111	408	0,579	2,577	-2,15
CDKN1C	ENSG00000129757	ENST00000440480	chr11:2904442-2907111	840	0,258	5,560	-4,43
CDKN1C	ENSG00000129757	ENST00000440480	chr11:2904442-2907111	192	0,278	5,560	-4,32
CDKN1C	ENSG00000129757	ENST00000440480	chr11:2904442-2907111	460	1,970	5,560	-1,50
CDKN1C	ENSG00000129757	ENST00000440480	chr11:2904442-2907111	346	1,386	5,560	-2,00
CDKN1C	ENSG00000129757	ENST00000440480	chr11:2904442-2907111	521	0,527	5,560	-3,40
CDKN1C	ENSG00000129757	ENST00000440480	chr11:2904442-2907111	238	0,205	5,560	-4,76
CDKN1C	ENSG00000129757	ENST00000440480	chr11:2904442-2907111	554	1,590	5,560	-1,81
CDKN1C	ENSG00000129757	ENST00000440480	chr11:2904442-2907111	408	0,260	5,560	-4,42
E2F1	ENSG00000101412	ENST00000343380	chr20:32263488-32274210	840	37,098	11,766	1,66
E2F1	ENSG00000101412	ENST00000343380	chr20:32263488-32274210	192	32,386	11,766	1,46
E2F1	ENSG00000101412	ENST00000343380	chr20:32263488-32274210	460	25,223	11,766	1,10
E2F1	ENSG00000101412	ENST00000343380	chr20:32263488-32274210	346	48,739	11,766	2,05
E2F1	ENSG00000101412	ENST00000343380	chr20:32263488-32274210	521	37,583	11,766	1,68
E2F1	ENSG00000101412	ENST00000343380	chr20:32263488-32274210	238	16,158	11,766	0,46
E2F1	ENSG00000101412	ENST00000343380	chr20:32263488-32274210	554	29,140	11,766	1,31
E2F1	ENSG00000101412	ENST00000343380	chr20:32263488-32274210	408	12,157	11,766	0,05
TP53	ENSG00000141510	ENST00000269305	chr17:7565096-7606820	840	41,756	16,740	1,32
TP53	ENSG00000141510	ENST00000269305	chr17:7565096-7606820	192	10,228	16,740	-0,71
TP53	ENSG00000141510	ENST00000269305	chr17:7565096-7606820	460	3,909	16,740	-2,10
TP53	ENSG00000141510	ENST00000269305	chr17:7565096-7606820	346	0,020	16,740	-9,74
TP53	ENSG00000141510	ENST00000269305	chr17:7565096-7606820	521	26,398	16,740	0,66
TP53	ENSG00000141510	ENST00000269305	chr17:7565096-7606820	238	23,495	16,740	0,49
TP53	ENSG00000141510	ENST00000269305	chr17:7565096-7606820	554	40,587	16,740	1,28
TP53	ENSG00000141510	ENST00000269305	chr17:7565096-7606820	408	23,087	16,740	0,46
TP53	ENSG00000141510	ENST00000420246	chr17:7565096-7606820	840	1,140	0,581	0,97
TP53	ENSG00000141510	ENST00000420246	chr17:7565096-7606820	192	1,304	0,581	1,17
TP53	ENSG00000141510	ENST00000420246	chr17:7565096-7606820	460	0,796	0,581	0,45
TP53	ENSG00000141510	ENST00000420246	chr17:7565096-7606820	346	0,670	0,581	0,21
TP53	ENSG00000141510	ENST00000420246	chr17:7565096-7606820	521	3,643	0,581	2,65
TP53	ENSG00000141510	ENST00000420246	chr17:7565096-7606820	238	0,350	0,581	-0,73
TP53	ENSG00000141510	ENST00000420246	chr17:7565096-7606820	554	0,650	0,581	0,16
TP53	ENSG00000141510	ENST00000420246	chr17:7565096-7606820	408	0,288	0,581	-1,01
TP53	ENSG00000141510	ENST00000445888	chr17:7565096-7606820	840	23,758	16,599	0,52
TP53	ENSG00000141510	ENST00000445888	chr17:7565096-7606820	192	6,595	16,599	-1,33
TP53	ENSG00000141510	ENST00000445888	chr17:7565096-7606820	460	11,213	16,599	-0,57
TP53	ENSG00000141510	ENST00000445888	chr17:7565096-7606820	346	1,194	16,599	-3,80
TP53	ENSG00000141510	ENST00000445888	chr17:7565096-7606820	521	35,518	16,599	1,10
TP53	ENSG00000141510	ENST00000445888	chr17:7565096-7606820	238	11,457	16,599	-0,53
TP53	ENSG00000141510	ENST00000445888	chr17:7565096-7606820	554	20,177	16,599	0,28
TP53	ENSG00000141510	ENST00000445888	chr17:7565096-7606820	408	11,944	16,599	-0,47
TP53	ENSG00000141510	ENST00000504937	chr17:7565096-7606820	840	22,821	9,559	1,26
TP53	ENSG00000141510	ENST00000504937	chr17:7565096-7606820	192	60,176	9,559	2,65
TP53	ENSG00000141510	ENST00000504937	chr17:7565096-7606820	460	50,764	9,559	2,41
TP53	ENSG00000141510	ENST00000504937	chr17:7565096-7606820	346	91,511	9,559	3,26
TP53	ENSG00000141510	ENST00000504937	chr17:7565096-7606820	521	9,238	9,559	-0,05
TP53	ENSG00000141510	ENST00000504937	chr17:7565096-7606820	238	16,812	9,559	0,81
TP53	ENSG00000141510	ENST00000504937	chr17:7565096-7606820	554	16,005	9,559	0,74
TP53	ENSG00000141510	ENST00000504937	chr17:7565096-7606820	408	9,463	9,559	-0,01

Ensembl_gene_ID, Ensembl gene identifier; Ensembl_transcript_ID, Ensembl transcript isoform identifier; FPKM, Fragments per kilobase of transcript per million mapped fragments; log2FC, log2 Fold changes of expression levels between tumours and controls.
