# Supplementary material for: RNA-Seq reveals the existence of a CDKN1C-E2F1-TP53 axis that is altered in human T-cell lymphoblastic lymphomas
Source: BMC Cancer. 2018 Apr 16;18:430. doi: 10.1186/s12885-018-4304-y (PMC5902834; doi:10.1186/s12885-018-4304-y)
Supplement: Supplementary file 6 — Table S4. Relative expression of CDKN1C, E2F1 and TP53 analyzed by qrtRT-PCR. (PDF 88 kb) [file 12885_2018_4304_MOESM6_ESM.pdf]

Supplementary Table 4: Relative expression of CDKN1C, E2F1 and TP53 analyzed by qrtRT-PCR.

Sample Cohort	Sample	ENST	ENSG	Gene Symbol	Housekeeping	2^-DCt 2_control	2^-DCt 2_tumor	P value	Norm_1	Norm_2	log2FC
Exploratory cohort	554	ENSG00000129757	ENSG00000129757	CDKN1C	b-actin	3,320	0,270	0,000	1,000	0,081	-3,625
		ENSG00000101412	ENSG00000101412	E2F1	b2-microglobulin	0,460	8,540	0,000	1,000	18,560	4,214
		ENST00000269305; ENST00000445888	ENSG00000141510	TP53	b-actin	1,190	1,470	0,001	1,000	1,240	0,310
		ENST00000504937	ENSG00000141510	TP53	b2-microglobulin	0,370	38,120	0,001	1,000	103,020	6,686
	238	ENSG00000129757	ENSG00000129757	CDKN1C	b-actin	3,320	0,490	0,000	1,000	0,147	-2,766
		ENSG00000101412	ENSG00000101412	E2F1	b2-microglobulin	0,460	0,270	0,074	1,000	0,590	-0,761
		ENST00000269305; ENST00000445888	ENSG00000141510	TP53	b-actin	1,190	1,720	0,001	1,000	1,450	0,536
		ENST00000504937	ENSG00000141510	TP53	b2-microglobulin	0,370	0,200	0,062	1,000	0,540	-0,888
	408	ENSG00000129757	ENSG00000129757	CDKN1C	b-actin	3,320	0,480	0,000	1,000	0,144	-2,795
		ENSG00000101412	ENSG00000101412	E2F1	b2-microglobulin	0,460	0,340	0,178	1,000	0,740	-0,434
		ENST00000269305; ENST00000445888	ENSG00000141510	TP53	b-actin	1,190	1,210	0,939	1,000	1,020	0,028
		ENST00000504937	ENSG00000141510	TP53	b2-microglobulin	0,370	0,380	0,742	1,000	1,020	0,038
	192	ENSG00000129757	ENSG00000129757	CDKN1C	b-actin	3,320	0,080	0,000	1,000	0,024	-5,380
		ENSG00000101412	ENSG00000101412	E2F1	b2-microglobulin	0,460	1,570	0,000	1,000	3,410	1,769
		ENST00000269305; ENST00000445888	ENSG00000141510	TP53	b-actin	1,190	0,690	0,001	1,000	0,580	-0,780
		ENST00000504937	ENSG00000141510	TP53	b2-microglobulin	0,370	5,040	0,000	1,000	13,620	3,767
	460	ENSG00000129757	ENSG00000129757	CDKN1C	b-actin	3,320	0,290	0,000	1,000	0,087	-3,522
		ENSG00000101412	ENSG00000101412	E2F1	b2-microglobulin	0,460	0,880	0,025	1,000	1,910	0,933
		ENST00000269305; ENST00000445888	ENSG00000141510	TP53	b-actin	1,190	0,920	0,005	1,000	0,760	-0,395
		ENST00000504937	ENSG00000141510	TP53	b2-microglobulin	0,370	3,630	0,000	1,000	9,810	3,294
	346	ENSG00000129757	ENSG00000129757	CDKN1C	b-actin	3,320	0,150	0,000	1,000	0,045	-4,473
		ENSG00000101412	ENSG00000101412	E2F1	b2-microglobulin	0,460	3,160	0,001	1,000	6,870	2,780
		ENST00000269305; ENST00000445888	ENSG00000141510	TP53	b-actin	1,190	1,050	0,154	1,000	0,880	-0,184
		ENST00000504937	ENSG00000141510	TP53	b2-microglobulin	0,370	0,670	0,020	1,000	1,810	0,855
	840	ENSG00000129757	ENSG00000129757	CDKN1C	b-actin	3,320	0,210	0,000	1,000	0,063	-3,988
		ENSG00000101412	ENSG00000101412	E2F1	b2-microglobulin	0,460	0,990	0,010	1,000	2,150	1,104
		ENST00000269305; ENST00000445888	ENSG00000141510	TP53	b-actin	1,190	1,880	0,003	1,000	1,580	0,659
		ENST00000504937	ENSG00000141510	TP53	b2-microglobulin	0,370	1,050	0,000	1,000	2,837	1,505
Extended cohort	829	ENSG00000129757	ENSG00000129757	CDKN1C	b-actin	3,320	0,410	0,000	1,000	0,123	-3,023
		ENSG00000101412	ENSG00000101412	E2F1	b2-microglobulin	0,460	0,340	0,167	1,000	0,739	-0,436
		ENST00000269305; ENST00000445888	ENSG00000141510	TP53	b-actin	1,190	1,340	0,014	1,000	1,126	0,171
		ENST00000504937	ENSG00000141510	TP53	b2-microglobulin	0,370	0,250	0,009	1,000	0,675	-0,401
	188	ENSG00000129757	ENSG00000129757	CDKN1C	b-actin	3,320	0,350	0,000	1,000	0,105	-3,251
		ENSG00000101412	ENSG00000101412	E2F1	b2-microglobulin	0,460	0,950	0,003	1,000	2,065	1,046
		ENST00000269305; ENST00000445888	ENSG00000141510	TP53	b-actin	1,190	1,230	0,267	1,000	1,033	0,046
		ENST00000504937	ENSG00000141510	TP53	b2-microglobulin	0,370	1,950	0,001	1,000	5,270	2,397
	080	ENSG00000129757	ENSG00000129757	CDKN1C	b-actin	3,320	0,360	0,000	1,000	0,108	-3,210
		ENSG00000101412	ENSG00000101412	E2F1	b2-microglobulin	0,460	0,470	0,957	1,000	1,021	0,029
		ENST00000269305; ENST00000445888	ENSG00000141510	TP53	b-actin	1,190	0,750	0,000	1,000	0,630	-0,666
		ENST00000504937	ENSG00000141510	TP53	b2-microglobulin	0,370	1,090	0,000	1,000	2,945	1,558
	135	ENSG00000129757	ENSG00000129757	CDKN1C	b-actin	3,320	0,130	0,000	1,000	0,039	-5,107
		ENSG00000101412	ENSG00000101412	E2F1	b2-microglobulin	0,460	1,540	0,004	1,000	3,347	1,742
		ENST00000269305; ENST00000445888	ENSG00000141510	TP53	b-actin	1,190	0,970	0,002	1,000	0,815	-0,295
		ENST00000504937	ENSG00000141510	TP53	b2-microglobulin	0,370	0,570	0,002	1,000	1,540	0,622
	104	ENSG00000129757	ENSG00000129757	CDKN1C	b-actin	3,320	0,120	0,000	1,000	0,036	-4,975
		ENSG00000101412	ENSG00000101412	E2F1	b2-microglobulin	0,460	0,930	0,005	1,000	2,021	1,015
		ENST00000269305	ENSG00000141510	TP53	b-actin	1,190	4,070	0,000	1,000	3,420	1,773
		ENST00000504937	ENSG00000141510	TP53	b2-microglobulin	0,370	11,170	0,000	1,000	30,189	4,915
	038	ENSG00000129757	ENSG00000129757	CDKN1C	b-actin	3,320	2,070	0,000	1,000	0,623	-0,682
		ENSG00000101412	ENSG00000101412	E2F1	b2-microglobulin	0,460	1,100	0,001	1,000	2,391	1,257
		ENST00000269305; ENST00000445888	ENSG00000141510	TP53	b-actin	1,190	1,710	0,000	1,000	1,436	0,522
		ENST00000504937	ENSG00000141510	TP53	b2-microglobulin	0,370	0,230	0,006	1,000	0,621	-0,687
	101	ENSG00000129757	ENSG00000129757	CDKN1C	b-actin	3,320	0,250	0,000	1,000	0,075	-3,736
		ENSG00000101412	ENSG00000101412	E2F1	b2-microglobulin	0,460	1,500	0,002	1,000	3,260	1,704
		ENST00000269305; ENST00000445888	ENSG00000141510	TP53	b-actin	1,190	0,370	0,000	1,000	0,310	-1,689
		ENST00000504937	ENSG00000141510	TP53	b2-microglobulin	0,370	32,310	0,001	1,000	87,320	6,448
	209	ENSG00000129757	ENSG00000129757	CDKN1C	b-actin	3,320	1,460	0,000	1,000	0,439	-1,187
		ENSG00000101412	ENSG00000101412	E2F1	b2-microglobulin	0,460	0,630	0,094	1,000	1,369	0,453
		ENST00000269305; ENST00000445888	ENSG00000141510	TP53	b-actin	1,190	1,560	0,002	1,000	1,310	0,389
		ENST00000504937	ENSG00000141510	TP53	b2-microglobulin	0,370	1,770	0,000	1,000	4,783	2,257
	639	ENSG00000129757	ENSG00000129757	CDKN1C	b-actin	3,320	0,590	0,000	1,000	0,177	-2,498
		ENSG00000101412	ENSG00000101412	E2F1	b2-microglobulin	0,460	0,330	0,143	1,000	0,717	-0,479
		ENST00000269305; ENST00000445888	ENSG00000141510	TP53	b-actin	1,190	1,250	0,099	1,000	1,050	0,070
		ENST00000504937	ENSG00000141510	TP53	b2-microglobulin	0,370	3,760	0,000	1,000	10,160	3,344
	001	ENSG00000129757	ENSG00000129757	CDKN1C	b-actin	3,320	0,490	0,000	1,000	0,147	-2,766
		ENSG00000101412	ENSG00000101412	E2F1	b2-microglobulin	0,460	2,890	0,006	1,000	6,282	2,651
		ENST00000269305; ENST00000445888	ENSG00000141510	TP53	b-actin	1,190	1,740	0,000	1,000	1,462	0,547
		ENST00000504937	ENSG00000141510	TP53	b2-microglobulin	0,370	1,470	0,000	1,000	3,972	1,989

Relative expression values were calculated as the mRNA amount of each gene relative to that of either β-actin or β2 microglobulin (used as reference) and normalized to the relative expression of normal control samples. 2^-DCt 2 numbers indicate mean expression values analysed by the 2^DDCt method obtained from three independent experiments in foetal thymuses and each tumour sample. Norm_1 and Norm_2 values represent normalized 2^-DCt 2 mean values. log2FC denotes the log-transformed (base 2) fold change normalized value of expression in each tumour sample relative to controls (foetal thymuses).
