# Supplementary material for: RNA-Seq reveals the existence of a CDKN1C-E2F1-TP53 axis that is altered in human T-cell lymphoblastic lymphomas
Source: BMC Cancer. 2018 Apr 16;18:430. doi: 10.1186/s12885-018-4304-y (PMC5902834; doi:10.1186/s12885-018-4304-y)
Supplement: Supplementary file 7 — Table S5. Complete list of genetic variants for TP53 gene determined by targeted deep sequencing in the T-LBL samples. (PDF 96 kb) [file 12885_2018_4304_MOESM7_ESM.pdf]

Supplementary Table 5 .- Complete list of genetic variants for TP53 gene determined by targeted deep sequencing in the T-LBL samples.

554

SIFT	PolyPhen2	LRT	MutationTaster	MutationAssessor	FATHMM	MetaSVM	Occurrences	HGMD	PROVEAN	# locus	type	ref	length	genotype	pvalue	coverage	allele_coverage	maf	gene	transcript	location	function	codon	exon	protein	coding	sift	polyphen	grantham	normalizedAlt	5000Exomes	cosmic	dbsnp	drugbank	go	omim	pfam	phylop
-	-	-	-	-	-	-	3	17 7577407 3055 A C	17,7577407,A,C	chr17:7577407	SNV	A	1	A/C	0.0	1525	852,673	0.164	TP53	NM_000546.5	intronic												rs12951053	1-(9-ethyl-9H-carbazol-3-yl)-N-methylmethanamine	GO:0005515:GO:0030308:GO:0003700:GO:0044212:GO:0009303:GO:0050731:GO:0009792:GO:0030154:GO:0034644:GO:0044419:GO:0002326:GO:0010332:GO:0046677:GO:0031625:GO:0000060:GO:0035035:GO:0035033:GO:0046982:GO:0005507:GO:0070245:GO:0007275:GO:0001701:GO:0071479:GO:0000733:GO:0042771:GO:0006284:GO:0000075:GO:0008270:GO:0006289:GO:0090399:GO:0000739:GO:0008134:GO:0005783:GO:0051087:GO:0048147:GO:0005634:GO:0001836:GO:0090343:GO:0008340:GO:0046872:GO:0016363:GO:0006302:GO:0005829:GO:0002309:GO:0051726:GO:0051721:GO:0046902:GO:0071456:GO:0042981:GO:0051097:GO:0005626:GO:0035690:GO:0006974:GO:0031065:GO:0007596:GO:0008156:GO:0031571:GO:0000790:GO:0006983:GO:0005657:GO:0001756:GO:0002020:GO:0071158:GO:0007406:GO:0042802:GO:0043525:GO:0002360:GO:0006978:GO:0000785:GO:0006977:GO:0033077:GO:0019901:GO:0030330:GO:0007569:GO:0000122:GO:0007417:GO:0043234:GO:0072332:GO:0005654:GO:0019899:GO:0045893:GO:2000379:GO:0045892:GO:0051276:GO:0007369:GO:0005678:GO:0008285:GO:0010165:GO:0008283:GO:0090403:GO:0007265:GO:0005739:GO:0002039:GO:0005737:GO:0008104:GO:0035264:GO:0045944:GO:0001085:GO:0007050:GO:0042149:GO:0030512:GO:0006355:GO:0043066:GO:0051262:GO:0005669:GO:0008635:GO:0043065:GO:0005730:GO:0006915:GO:0003677:GO:0005524:GO:0003684:GO:0016604:GO:0070215:GO:0047485:GO:0006461:GO:0003682:GO:0016605:GO:0034613:GO:0008629	191170	PF08563:PF07710:PF00870	-1.68
-	-	-	-	-	-	-	3	17 7577427 3056 G A	17,7577427,G,A	chr17:7577427	SNV	G	1	G/A	0.0	1535	873,662	0.164	TP53	NM_000546.5	intronic												rs12947788	1-(9-ethyl-9H-carbazol-3-yl)-N-methylmethanamine	GO:0005515:GO:0030308:GO:0003700:GO:0044212:GO:0009303:GO:0050731:GO:0009792:GO:0030154:GO:0034644:GO:0044419:GO:0002326:GO:0010332:GO:0046677:GO:0031625:GO:0000060:GO:0035035:GO:0035033:GO:0046982:GO:0005507:GO:0070245:GO:0007275:GO:0001701:GO:0071479:GO:0000733:GO:0042771:GO:0006284:GO:0000075:GO:0008270:GO:0006289:GO:0090399:GO:0000739:GO:0008134:GO:0005783:GO:0051087:GO:0048147:GO:0005634:GO:0001836:GO:0090343:GO:0008340:GO:0046872:GO:0016363:GO:0006302:GO:0005829:GO:0002309:GO:0051726:GO:0051721:GO:0046902:GO:0071456:GO:0042981:GO:0051097:GO:0005626:GO:0035690:GO:0006974:GO:0031065:GO:0007596:GO:0008156:GO:0031571:GO:0000790:GO:0006983:GO:0005657:GO:0001756:GO:0002020:GO:0071158:GO:0007406:GO:0042802:GO:0043525:GO:0002360:GO:0006978:GO:0000785:GO:0006977:GO:0033077:GO:0019901:GO:0030330:GO:0007569:GO:0000122:GO:0007417:GO:0043234:GO:0072332:GO:0005654:GO:0019899:GO:0045893:GO:2000379:GO:0045892:GO:0051276:GO:0007369:GO:0005678:GO:0008285:GO:0010165:GO:0008283:GO:0090403:GO:0007265:GO:0005739:GO:0002039:GO:0005737:GO:0008104:GO:0035264:GO:0045944:GO:0001085:GO:0007050:GO:0042149:GO:0030512:GO:0006355:GO:0043066:GO:0051262:GO:0005669:GO:0008635:GO:0043065:GO:0005730:GO:0006915:GO:0003677:GO:0005524:GO:0003684:GO:0016604:GO:0070215:GO:0047485:GO:0006461:GO:0003682:GO:0016605:GO:0034613:GO:0008629	191170	PF08563:PF07710:PF00870	0.16
-	-	-	-	-	-	-	0	17 7579470 3448 CGG CGC	17,7579470,CGG,CGC	chr17:7579470	SNV	CGG	1	CGG/CGC	0.0	583	296,287,0,0,0	0.398:0.0	TP53	NM_000546.5	exonic	missense	CGC	4	p.Pro72Arg	c.215C>G			103.0	C		43787:18569:1268332:1268331:96586:45288:46307:44256:44466:1386925:1386926:46058:46408	rs1042522:rs56275308	1-(9-ethyl-9H-carbazol-3-yl)-N-methylmethanamine	GO:0005515:GO:0030308:GO:0003700:GO:0044212:GO:0009303:GO:0050731:GO:0009792:GO:0030154:GO:0034644:GO:0044419:GO:0002326:GO:0010332:GO:0046677:GO:0031625:GO:0000060:GO:0035035:GO:0035033:GO:0046982:GO:0005507:GO:0070245:GO:0007275:GO:0001701:GO:0071479:GO:0000733:GO:0042771:GO:0006284:GO:0000075:GO:0008270:GO:0006289:GO:0090399:GO:0000739:GO:0008134:GO:0005783:GO:0051087:GO:0048147:GO:0005634:GO:0001836:GO:0090343:GO:0008340:GO:0046872:GO:0016363:GO:0006302:GO:0005829:GO:0002309:GO:0051726:GO:0051721:GO:0046902:GO:0071456:GO:0042981:GO:0051097:GO:0005626:GO:0035690:GO:0006974:GO:0031065:GO:0007596:GO:0008156:GO:0031571:GO:0000790:GO:0006983:GO:0005657:GO:0001756:GO:0002020:GO:0071158:GO:0007406:GO:0042802:GO:0043525:GO:0002360:GO:0006978:GO:0000785:GO:0006977:GO:0033077:GO:0019901:GO:0030330:GO:0007569:GO:0000122:GO:0007417:GO:0043234:GO:0072332:GO:0005654:GO:0019899:GO:0045893:GO:2000379:GO:0045892:GO:0051276:GO:0007369:GO:0005678:GO:0008285:GO:0010165:GO:0008283:GO:0090403:GO:0007265:GO:0005739:GO:0002039:GO:0005737:GO:0008104:GO:0035264:GO:0045944:GO:0001085:GO:0007050:GO:0042149:GO:0030512:GO:0006355:GO:0043066:GO:0051262:GO:0005669:GO:0008635:GO:0043065:GO:0005730:GO:0006915:GO:0003677:GO:0005524:GO:0003684:GO:0016604:GO:0070215:GO:0047485:GO:0006461:GO:0003682:GO:0016605:GO:0034613:GO:0008629	191170	PF08563:PF07710:PF00870	-1.14,-0.69,1.36

238

SIFT	PolyPhen2	LRT	MutationTaster	MutationAssessor	FATHMM	MetaSVM	Occurrences	HGMD	PROVEAN	# locus	type	ref	length	genotype	pvalue	coverage	allele_coverage	maf	gene	transcript	location	function	codon	exon	protein	coding	sift	polyphen	grantham	normalizedAlt	5000Exomes	cosmic	dbsnp	drugbank	go	omim	pfam	phylop
-	-	-	-	-	-	-	0	17 7579470 3416 CGG CGC	17,7579470,CGG,CGC	chr17:7579470	SNV	CGG	1	CGG/CGC	0.0	761	31,750,0,0,0	0.398:0.0	TP53	NM_000546.5	exonic	missense	CGC	4	p.Pro72Arg	c.215C>G			103.0	C		43787:18569:1268332:1268331:96586:45288:46307:44256:44466:1386925:1386926:46058:46408	rs1042522:rs56275308	1-(9-ethyl-9H-carbazol-3-yl)-N-methylmethanamine	GO:0005515:GO:0030308:GO:0003700:GO:0044212:GO:0009303:GO:0050731:GO:0009792:GO:0030154:GO:0034644:GO:0044419:GO:0002326:GO:0010332:GO:0046677:GO:0031625:GO:0000060:GO:0035035:GO:0035033:GO:0046982:GO:0005507:GO:0070245:GO:0007275:GO:0001701:GO:0071479:GO:0000733:GO:0042771:GO:0006284:GO:0000075:GO:0008270:GO:0006289:GO:0090399:GO:0000739:GO:0008134:GO:0005783:GO:0051087:GO:0048147:GO:0005634:GO:0001836:GO:0090343:GO:0008340:GO:0046872:GO:0016363:GO:0006302:GO:0005829:GO:0002309:GO:0051726:GO:0051721:GO:0046902:GO:0071456:GO:0042981:GO:0051097:GO:0005626:GO:0035690:GO:0006974:GO:0031065:GO:0007596:GO:0008156:GO:0031571:GO:0000790:GO:0006983:GO:0005657:GO:0001756:GO:0002020:GO:0071158:GO:0007406:GO:0042802:GO:0043525:GO:0002360:GO:0006978:GO:0000785:GO:0006977:GO:0033077:GO:0019901:GO:0030330:GO:0007569:GO:0000122:GO:0007417:GO:0043234:GO:0072332:GO:0005654:GO:0019899:GO:0045893:GO:2000379:GO:0045892:GO:0051276:GO:0007369:GO:0005678:GO:0008285:GO:0010165:GO:0008283:GO:0090403:GO:0007265:GO:0005739:GO:0002039:GO:0005737:GO:0008104:GO:0035264:GO:0045944:GO:0001085:GO:0007050:GO:0042149:GO:0030512:GO:0006355:GO:0043066:GO:0051262:GO:0005669:GO:0008635:GO:0043065:GO:0005730:GO:0006915:GO:0003677:GO:0005524:GO:0003684:GO:0016604:GO:0070215:GO:0047485:GO:0006461:GO:0003682:GO:0016605:GO:0034613:GO:0008629	191170	PF08563:PF07710:PF00870	-1.14,-0.69,1.36

408

SIFT	PolyPhen2	LRT	MutationTaster	MutationAssessor	FATHMM	MetaSVM	Occurrences	HGMD	PROVEAN	# locus	type	ref	length	genotype	pvalue	coverage	allele_coverage	maf	gene	transcript	location	function	codon	exon	protein	coding	sift	polyphen	grantham	normalizedAlt	5000Exomes	cosmic	dbsnp	drugbank	go	omim	pfam	phylop
-	-	-	-	-	-	-	2	17 7579619 3437 G T	17,7579619,G,T	chr17:7579619	SNV	G	1	G/T	0.0	1232	629,603	0.073	TP53	NM_000546.5	intronic										AMAF=0.1067:EMAF=0.0524:GMAF=0.0708		rs17883323	1-(9-ethyl-9H-carbazol-3-yl)-N-methylmethanamine	GO:0005515:GO:0030308:GO:0003700:GO:0044212:GO:0009303:GO:0050731:GO:0009792:GO:0030154:GO:0034644:GO:0044419:GO:0002326:GO:0010332:GO:0046677:GO:0031625:GO:0000060:GO:0035035:GO:0035033:GO:0046982:GO:0005507:GO:0070245:GO:0007275:GO:0001701:GO:0071479:GO:0000733:GO:0042771:GO:0006284:GO:0000075:GO:0008270:GO:0006289:GO:0090399:GO:0000739:GO:0008134:GO:0005783:GO:0051087:GO:0048147:GO:0005634:GO:0001836:GO:0090343:GO:0008340:GO:0046872:GO:0016363:GO:0006302:GO:0005829:GO:0002309:GO:0051726:GO:0051721:GO:0046902:GO:0071456:GO:0042981:GO:0051097:GO:0005626:GO:0035690:GO:0006974:GO:0031065:GO:0007596:GO:0008156:GO:0031571:GO:0000790:GO:0006983:GO:0005657:GO:0001756:GO:0002020:GO:0071158:GO:0007406:GO:0042802:GO:0043525:GO:0002360:GO:0006978:GO:0000785:GO:0006977:GO:0033077:GO:0019901:GO:0030330:GO:0007569:GO:0000122:GO:0007417:GO:0043234:GO:0072332:GO:0005654:GO:0019899:GO:0045893:GO:2000379:GO:0045892:GO:0051276:GO:0007369:GO:0005678:GO:0008285:GO:0010165:GO:0008283:GO:0090403:GO:0007265:GO:0005739:GO:0002039:GO:0005737:GO:0008104:GO:0035264:GO:0045944:GO:0001085:GO:0007050:GO:0042149:GO:0030512:GO:0006355:GO:0043066:GO:0051262:GO:0005669:GO:0008635:GO:0043065:GO:0005730:GO:0006915:GO:0003677:GO:0005524:GO:0003684:GO:0016604:GO:0070215:GO:0047485:GO:0006461:GO:0003682:GO:0016605:GO:0034613:GO:0008629	191170	PF08563:PF07710:PF00870	-0.7
-	-	-	-	-	-	-	12	17 7579643 3438 CCCCCAGCCCTCCAGGT C	17,7579643,CCCCCAGCCCTCCAGGT,C	chr17:7579643	INDEL	CCCCCAGCCCTCCAGGT	16	CCCCCAGCCCTCCAGGT/C	1,00E+10	2417	2007,41	0.153	TP53	NM_000546.5	intronic										AMAF=0.3385:EMAF=0.2049:GMAF=0.2511		rs59758982:rs17878362:rs150200764:rs146534833:rs139100308:rs67112752:rs66510636:rs138066738	1-(9-ethyl-9H-carbazol-3-yl)-N-methylmethanamine	GO:0005515:GO:0030308:GO:0003700:GO:0044212:GO:0009303:GO:0050731:GO:0009792:GO:0030154:GO:0034644:GO:0044419:GO:0002326:GO:0010332:GO:0046677:GO:0031625:GO:0000060:GO:0035035:GO:0035033:GO:0046982:GO:0005507:GO:0070245:GO:0007275:GO:0001701:GO:0071479:GO:0000733:GO:0042771:GO:0006284:GO:0000075:GO:0008270:GO:0006289:GO:0090399:GO:0000739:GO:0008134:GO:0005783:GO:0051087:GO:0048147:GO:0005634:GO:0001836:GO:0090343:GO:0008340:GO:0046872:GO:0016363:GO:0006302:GO:0005829:GO:0002309:GO:0051726:GO:0051721:GO:0046902:GO:0071456:GO:0042981:GO:0051097:GO:0005626:GO:0035690:GO:0006974:GO:0031065:GO:0007596:GO:0008156:GO:0031571:GO:0000790:GO:0006983:GO:0005657:GO:0001756:GO:0002020:GO:0071158:GO:0007406:GO:0042802:GO:0043525:GO:0002360:GO:0006978:GO:0000785:GO:0006977:GO:0033077:GO:0019901:GO:0030330:GO:0007569:GO:0000122:GO:0007417:GO:0043234:GO:0072332:GO:0005654:GO:0019899:GO:0045893:GO:2000379:GO:0045892:GO:0051276:GO:0007369:GO:0005678:GO:0008285:GO:0010165:GO:0008283:GO:0090403:GO:0007265:GO:0005739:GO:0002039:GO:0005737:GO:0008104:GO:0035264:GO:0045944:GO:0001085:GO:0007050:GO:0042149:GO:0030512:GO:0006355:GO:0043066:GO:0051262:GO:0005669:GO:0008635:GO:0043065:GO:0005730:GO:0006915:GO:0003677:GO:0005524:GO:0003684:GO:0016604:GO:0070215:GO:0047485:GO:0006461:GO:0003682:GO:0016605:GO:0034613:GO:0008629	191170	PF08563:PF07710:PF00870	2.36,0.13,-0.88,0.2,0.15,-0.39,-1.0,-1.96,-1.18,0.06,0.06,0.46,0.46,0.38,-0.47,0.46,0.38

192

SIFT	PolyPhen2	LRT	MutationTaster	MutationAssessor	FATHMM	MetaSVM	Occurrences	HGMD	PROVEAN	# locus	type	ref	length	genotype	pvalue	coverage	allele_coverage	maf	gene	transcript	location	function	codon	exon	protein	coding	sift	polyphen	grantham	normalizedAlt	5000Exomes	cosmic	dbsnp	drugbank	go	omim	pfam	phylop
D	B	.	N	.	.	T	0	17 7578146 3302 T C	17,7578146,T,C	chr17:7578146	SNV	T	1	T/C	3,83E-08	77	49,24	0.0	TP53	NM_000546.5	intronic										AMAF=9.0E-4:EMAF=0.0051:GMAF=0.0037	45453	rs34949160	1-(9-ethyl-9H-carbazol-3-yl)-N-methylmethanamine	GO:0005515:GO:0030308:GO:0003700:GO:0044212:GO:0009303:GO:0050731:GO:0009792:GO:0030154:GO:0034644:GO:0044419:GO:0002326:GO:0010332:GO:0046677:GO:0031625:GO:0000060:GO:0035035:GO:0035033:GO:0046982:GO:0005507:GO:0070245:GO:0007275:GO:0001701:GO:0071479:GO:0000733:GO:0042771:GO:0006284:GO:0000075:GO:0008270:GO:0006289:GO:0090399:GO:0000739:GO:0008134:GO:0005783:GO:0051087:GO:0048147:GO:0005634:GO:0001836:GO:0090343:GO:0008340:GO:0046872:GO:0016363:GO:0006302:GO:0005829:GO:0002309:GO:0051726:GO:0051721:GO:0046902:GO:0071456:GO:0042981:GO:0051097:GO:0005626:GO:0035690:GO:0006974:GO:0031065:GO:0007596:GO:0008156:GO:0031571:GO:0000790:GO:0006983:GO:0005657:GO:0001756:GO:0002020:GO:0071158:GO:0007406:GO:0042802:GO:0043525:GO:0002360:GO:0006978:GO:0000785:GO:0006977:GO:0033077:GO:0019901:GO:0030330:GO:0007569:GO:0000122:GO:0007417:GO:0043234:GO:0072332:GO:0005654:GO:0019899:GO:0045893:GO:2000379:GO:0045892:GO:0051276:GO:0007369:GO:0005678:GO:0008285:GO:0010165:GO:0008283:GO:0090403:GO:0007265:GO:0005739:GO:0002039:GO:0005737:GO:0008104:GO:0035264:GO:0045944:GO:0001085:GO:0007050:GO:0042149:GO:0030512:GO:0006355:GO:0043066:GO:0051262:GO:0005669:GO:0008635:GO:0043065:GO:0005730:GO:0006915:GO:0003677:GO:0005524:GO:0003684:GO:0016604:GO:0070215:GO:0047485:GO:0006461:GO:0003682:GO:0016605:GO:0034613:GO:0008629	191170	PF08563:PF07710:PF00870	-0.3
T	P	N	D	M	D	D	0	17 7578503 3509 C A	17,7578503,C,A	chr17:7578503	SNV	C	1	C/A	0.1733803997754138	140	137,3,0		TP53	NM_000546.5	exonic	missense	TTG	5	p.Val143Leu	c.427G>T	0.12	0.401	32.0	A		44904:46078:45958:220756:1664639:220753:44576:220755:220754:46033:43878		1-(9-ethyl-9H-carbazol-3-yl)-N-methylmethanamine	GO:0005515:GO:0030308:GO:0003700:GO:0044212:GO:0009303:GO:0050731:GO:0009792:GO:0030154:GO:0034644:GO:0044419:GO:0002326:GO:0010332:GO:0046677:GO:0031625:GO:0000060:GO:0035035:GO:0035033:GO:0046982:GO:0005507:GO:0070245:GO:0007275:GO:0001701:GO:0071479:GO:0000733:GO:0042771:GO:0006284:GO:0000075:GO:0008270:GO:0006289:GO:0090399:GO:0000739:GO:0008134:GO:0005783:GO:0051087:GO:0048147:GO:0005634:GO:0001836:GO:0090343:GO:0008340:GO:0046872:GO:0016363:GO:0006302:GO:0005829:GO:0002309:GO:0051726:GO:0051721:GO:0046902:GO:0071456:GO:0042981:GO:0051097:GO:0005626:GO:0035690:GO:0006974:GO:0031065:GO:0007596:GO:0008156:GO:0031571:GO:0000790:GO:0006983:GO:0005657:GO:0001756:GO:0002020:GO:0071158:GO:0007406:GO:0042802:GO:0043525:GO:0002360:GO:0006978:GO:0000785:GO:0006977:GO:0033077:GO:0019901:GO:0030330:GO:0007569:GO:0000122:GO:0007417:GO:0043234:GO:0072332:GO:0005654:GO:0019899:GO:0045893:GO:2000379:GO:0045892:GO:0051276:GO:0007369:GO:0005678:GO:0008285:GO:0010165:GO:0008283:GO:0090403:GO:0007265:GO:0005739:GO:0002039:GO:0005737:GO:0008104:GO:0035264:GO:0045944:GO:0001085:GO:0007050:GO:0042149:GO:0030512:GO:0006355:GO:0043066:GO:0051262:GO:0005669:GO:0008635:GO:0043065:GO:0005730:GO:0006915:GO:0003677:GO:0005524:GO:0003684:GO:0016604:GO:0070215:GO:0047485:GO:0006461:GO:0003682:GO:0016605:GO:0034613:GO:0008629	191170	PF08563:PF07710:PF00870	1.45
-	-	-	-	-	-	-	0	17 7579470 3601 CGG CGC	17,7579470,CGG,CGC	chr17:7579470	SNV	CGG	1	CGG/CGC	5,15E-85	153	69,81,3,0,0	0.398:0.0	TP53	NM_000546.5	exonic	missense	CGC	4	p.Pro72Arg	c.215C>G			103.0	C		43787:18569:1268332:1268331:96586:45288:46307:44256:44466:1386925:1386926:46058:46408	rs1042522:rs56275308	1-(9-ethyl-9H-carbazol-3-yl)-N-methylmethanamine	GO:0005515:GO:0030308:GO:0003700:GO:0044212:GO:0009303:GO:0050731:GO:0009792:GO:0030154:GO:0034644:GO:0044419:GO:0002326:GO:0010332:GO:0046677:GO:0031625:GO:0000060:GO:0035035:GO:0035033:GO:0046982:GO:0005507:GO:0070245:GO:0007275:GO:0001701:GO:0071479:GO:0000733:GO:0042771:GO:0006284:GO:0000075:GO:0008270:GO:0006289:GO:0090399:GO:0000739:GO:0008134:GO:0005783:GO:0051087:GO:0048147:GO:0005634:GO:0001836:GO:0090343:GO:0008340:GO:0046872:GO:0016363:GO:0006302:GO:0005829:GO:0002309:GO:0051726:GO:0051721:GO:0046902:GO:0071456:GO:0042981:GO:0051097:GO:0005626:GO:0035690:GO:0006974:GO:0031065:GO:0007596:GO:0008156:GO:0031571:GO:0000790:GO:0006983:GO:0005657:GO:0001756:GO:0002020:GO:0071158:GO:0007406:GO:0042802:GO:0043525:GO:0002360:GO:0006978:GO:0000785:GO:0006977:GO:0033077:GO:0019901:GO:0030330:GO:0007569:GO:0000122:GO:0007417:GO:0043234:GO:0072332:GO:0005654:GO:0019899:GO:0045893:GO:2000379:GO:0045892:GO:0051276:GO:0007369:GO:0005678:GO:0008285:GO:0010165:GO:0008283:GO:0090403:GO:0007265:GO:0005739:GO:0002039:GO:0005737:GO:0008104:GO:0035264:GO:0045944:GO:0001085:GO:0007050:GO:0042149:GO:0030512:GO:0006355:GO:0043066:GO:0051262:GO:0005669:GO:0008635:GO:0043065:GO:0005730:GO:0006915:GO:0003677:GO:0005524:GO:0003684:GO:0016604:GO:0070215:GO:0047485:GO:0006461:GO:0003682:GO:0016605:GO:0034613:GO:0008629	191170	PF08563:PF07710:PF00870	-1.14,-0.69,1.36

-	-	-	-	-	-	-	0	17 7579643 3630 CCCCCAGCCCTCCAGGT CCCCAGCCCCTCCAGGT	17,7579643,CCCCCAGCCCTCCAGGT,CCCCAGCCCCTCCAGGT	chr17:7579643	MNV,INDEL	CCCCCAGCCCTCCAGGT	16	C/CCCCAGCCCCTCCAGGT	3,74E-106	212	51,71,0,90	0.153	TP53	NM_000546.5	intronic										AMAF=0.3385:EMAF=0.2049:GMAF=0.2511		rs59758982:rs17878362:rs150200764:rs146534833:rs139100308:rs67112752:rs66510636:rs138066738	1-(9-ethyl-9H-carbazol-3-yl)-N-methylmethanamine	GO:0005515:GO:0030308:GO:0003700:GO:0044212:GO:0009303:GO:0050731:GO:0009792:GO:0030154:GO:0034644:GO:0044419:GO:0002326:GO:0010332:GO:0046677:GO:0031625:GO:0000060:GO:0035035:GO:0035033:GO:0046982:GO:0005507:GO:0070245:GO:0007275:GO:0001701:GO:0071479:GO:0000733:GO:0042771:GO:0006284:GO:0000075:GO:0008270:GO:0006289:GO:0090399:GO:0000739:GO:0008134:GO:0005783:GO:0051087:GO:0048147:GO:0005634:GO:0001836:GO:0090343:GO:0008340:GO:0046872:GO:0016363:GO:0006302:GO:0005829:GO:0002309:GO:0051726:GO:0051721:GO:0046902:GO:0071456:GO:0042981:GO:0051097:GO:0005626:GO:0035690:GO:0006974:GO:0031065:GO:0007596:GO:0008156:GO:0031571:GO:0000790:GO:0006983:GO:0005657:GO:0001756:GO:0002020:GO:0071158:GO:0007406:GO:0042802:GO:0043525:GO:0002360:GO:0006978:GO:0000785:GO:0006977:GO:0033077:GO:0019901:GO:0030330:GO:0007569:GO:0000122:GO:0007417:GO:0043234:GO:0072332:GO:0005654:GO:0019899:GO:0045893:GO:2000379:GO:0045892:GO:0051276:GO:0007369:GO:0005678:GO:0008285:GO:0010165:GO:0008283:GO:0090403:GO:0007265:GO:0005739:GO:0002039:GO:0005737:GO:0008104:GO:0035264:GO:0045944:GO:0001085:GO:0007050:GO:0042149:GO:0030512:GO:0006355:GO:0043066:GO:0051262:GO:0005669:GO:0008635:GO:0043065:GO:0005730:GO:0006915:GO:0003677:[illegible]	191170	PF08563:PF07710:PF00870	2.36,0.13,-0.88,0.2,0.15,-0.39,-1.0,-1.96,-1.18,0.06,0.06,0.46,0.46,0.38,-0.47,0.46,0.38

460

# locus	type	ref	length	genotype	pvalue	coverage	allele_coverage	maf	gene	transcript	location	function	codon	exon	protein	coding	sift	polyphen	grantham	normalizedAlt	5000Exomes	cosmic	dbsnp	drugbank	go	omim	pfam	phylop
chr17:7576841	SNV	A	1	A/G	0.0	1999	1002,997	0.017	TP53	NM_000546.5	intronic										AMAF=0.0272:EMAF=0.0147:GMAF=0.0189		rs1800899	1-(9-ethyl-9H-carbazol-3-yl)-N-methylmethanamine	GO:0005515:GO:0030308:GO:0003700:GO:0044212:GO:0009303:GO:0050731:GO:0009792:GO:0030154:GO:0034644:GO:0044419:GO:0002326:GO:0010332:GO:0046677:GO:0031625:GO:0000060:GO:0035035:GO:0035033:GO:0046982:GO:0005507:GO:0070245:GO:0007275:GO:0001701:GO:0071479:GO:0000733:GO:0042771:GO:0006284:GO:0000075:GO:0008270:GO:0006289:GO:0090399:GO:0000739:GO:0008134:GO:0005783:GO:0051087:GO:0048147:GO:0005634:GO:0001836:GO:0090343:GO:0008340:GO:0046872:GO:0016363:GO:0006302:GO:0005829:GO:0002309:GO:0051726:GO:0051721:GO:0046902:GO:0071456:GO:0042981:GO:0051097:GO:0005626:GO:0035690:GO:0006974:GO:0031065:GO:0007596:GO:0008156:GO:0031571:GO:0000790:GO:0006983:GO:0005657:GO:0001756:GO:0002020:GO:0071158:GO:0007406:GO:0042802:GO:0043525:GO:0002360:GO:0006978:GO:0000785:GO:0006977:GO:0033077:GO:0019901:GO:0030330:GO:0007569:GO:0000122:GO:0007417:GO:0043234:GO:0072332:GO:0005654:GO:0019899:GO:0045893:GO:2000379:GO:0045892:GO:0051276:GO:0007369:GO:0005678:GO:0008285:GO:0010165:GO:0008283:GO:0090403:GO:0007265:GO:0005739:GO:0002039:GO:0005737:GO:0008104:GO:0035264:GO:0045944:GO:0001085:GO:0007050:GO:0042149:GO:0030512:GO:0006355:GO:0043066:GO:0051262:GO:0005669:GO:0008635:GO:0043065:GO:0005730:GO:0006915:GO:0003677:GO:0005524:GO:0003684:GO:0016604:GO:0070215:GO:0047485:GO:0006461:GO:0003682:GO:0016605:GO:0034613:GO:0008629	191170	PF08563:PF07710:PF00870	-1.12
chr17:7579470	SNV	CGG	1	CGG/CGC	0.0	982	403,517,0,62,0,0	0.398:0.0	TP53	NM_000546.5	exonic	missense	CGC	4	p.Pro72Arg	c.215C>G			103.0	C		43787:18569:1268332:1268331:96586:45288:46307:44256:44466:1386925:1386926:46058:46408	rs1042522:rs56275308	1-(9-ethyl-9H-carbazol-3-yl)-N-methylmethanamine	GO:0005515:GO:0030308:GO:0003700:GO:0044212:GO:0009303:GO:0050731:GO:0009792:GO:0030154:GO:0034644:GO:0044419:GO:0002326:GO:0010332:GO:0046677:GO:0031625:GO:0000060:GO:0035035:GO:0035033:GO:0046982:GO:0005507:GO:0070245:GO:0007275:GO:0001701:GO:0071479:GO:0000733:GO:0042771:GO:0006284:GO:0000075:GO:0008270:GO:0006289:GO:0090399:GO:0000739:GO:0008134:GO:0005783:GO:0051087:GO:0048147:GO:0005634:GO:0001836:GO:0090343:GO:0008340:GO:0046872:GO:0016363:GO:0006302:GO:0005829:GO:0002309:GO:0051726:GO:0051721:GO:0046902:GO:0071456:GO:0042981:GO:0051097:GO:0005626:GO:0035690:GO:0006974:GO:0031065:GO:0007596:GO:0008156:GO:0031571:GO:0000790:GO:0006983:GO:0005657:GO:0001756:GO:0002020:GO:0071158:GO:0007406:GO:0042802:GO:0043525:GO:0002360:GO:0006978:GO:0000785:GO:0006977:GO:0033077:GO:0019901:GO:0030330:GO:0007569:GO:0000122:GO:0007417:GO:0043234:GO:0072332:GO:0005654:GO:0019899:GO:0045893:GO:2000379:GO:0045892:GO:0051276:GO:0007369:GO:0005678:GO:0008285:GO:0010165:GO:0008283:GO:0090403:GO:0007265:GO:0005739:GO:0002039:GO:0005737:GO:0008104:GO:0035264:GO:0045944:GO:0001085:GO:0007050:GO:0042149:GO:0030512:GO:0006355:GO:0043066:GO:0051262:GO:0005669:GO:0008635:GO:0043065:GO:0005730:GO:0006915:GO:0003677:GO:0005524:GO:0003684:GO:0016604:GO:0070215:GO:0047485:GO:0006461:GO:0003682:GO:0016605:GO:0034613:GO:0008629	191170	PF08563:PF07710:PF00870	-1.14,-0.69,1.36
chr17:7579579	SNV	C	1	C/T	0.0	1599	794,805	0.012	TP53	NM_000546.5	exonic	synonymous	CCA	4	p.(=)	c.108G>A				T	AMAF=0.0045:EMAF=0.0148:GMAF=0.0113	53250	rs1800370	1-(9-ethyl-9H-carbazol-3-yl)-N-methylmethanamine	GO:0005515:GO:0030308:GO:0003700:GO:0044212:GO:0009303:GO:0050731:GO:0009792:GO:0030154:GO:0034644:GO:0044419:GO:0002326:GO:0010332:GO:0046677:GO:0031625:GO:0000060:GO:0035035:GO:0035033:GO:0046982:GO:0005507:GO:0070245:GO:0007275:GO:0001701:GO:0071479:GO:0000733:GO:0042771:GO:0006284:GO:0000075:GO:0008270:GO:0006289:GO:0090399:GO:0000739:GO:0008134:GO:0005783:GO:0051087:GO:0048147:GO:0005634:GO:0001836:GO:0090343:GO:0008340:GO:0046872:GO:0016363:GO:0006302:GO:0005829:GO:0002309:GO:0051726:GO:0051721:GO:0046902:GO:0071456:GO:0042981:GO:0051097:GO:0005626:GO:0035690:GO:0006974:GO:0031065:GO:0007596:GO:0008156:GO:0031571:GO:0000790:GO:0006983:GO:0005657:GO:0001756:GO:0002020:GO:0071158:GO:0007406:GO:0042802:GO:0043525:GO:0002360:GO:0006978:GO:0000785:GO:0006977:GO:0033077:GO:0019901:GO:0030330:GO:0007569:GO:0000122:GO:0007417:GO:0043234:GO:0072332:GO:0005654:GO:0019899:GO:0045893:GO:2000379:GO:0045892:GO:0051276:GO:0007369:GO:0005678:GO:0008285:GO:0010165:GO:0008283:GO:0090403:GO:0007265:GO:0005739:GO:0002039:GO:0005737:GO:0008104:GO:0035264:GO:0045944:GO:0001085:GO:0007050:GO:0042149:GO:0030512:GO:0006355:GO:0043066:GO:0051262:GO:0005669:GO:0008635:GO:0043065:GO:0005730:GO:0006915:GO:0003677:GO:0005524:GO:0003684:GO:0016604:GO:0070215:GO:0047485:GO:0006461:GO:0003682:GO:0016605:GO:0034613:GO:0008629	191170	PF08563:PF07710:PF00870	-1.05
chr17:7579643	INDEL,MNV	CCCCCAGCCCTCCAGGT	16	C/CCCCAGCCCCTCCAGGT	0.0	852	143,275,434	0.153	TP53	NM_000546.5	intronic										AMAF=0.3385:EMAF=0.2049:GMAF=0.2511		rs59758982:rs17878362:rs150200764:rs146534833:rs139100308:rs67112752:rs66510636:rs138066738	1-(9-ethyl-9H-carbazol-3-yl)-N-methylmethanamine	GO:0005515:GO:0030308:GO:0003700:GO:0044212:GO:0009303:GO:0050731:GO:0009792:GO:0030154:GO:0034644:GO:0044419:GO:0002326:GO:0010332:GO:0046677:GO:0031625:GO:0000060:GO:0035035:GO:0035033:GO:0046982:GO:0005507:GO:0070245:GO:0007275:GO:0001701:GO:0071479:GO:0000733:GO:0042771:GO:0006284:GO:0000075:GO:0008270:GO:0006289:GO:0090399:GO:0000739:GO:0008134:GO:0005783:GO:0051087:GO:0048147:GO:0005634:GO:0001836:GO:0090343:GO:0008340:GO:0046872:GO:0016363:GO:0006302:GO:0005829:GO:0002309:GO:0051726:GO:0051721:GO:0046902:GO:0071456:GO:0042981:GO:0051097:GO:0005626:GO:0035690:GO:0006974:GO:0031065:GO:0007596:GO:0008156:GO:0031571:GO:0000790:GO:0006983:GO:0005657:GO:0001756:GO:0002020:GO:0071158:GO:0007406:GO:0042802:GO:0043525:GO:0002360:GO:0006978:GO:0000785:GO:0006977:GO:0033077:GO:0019901:GO:0030330:GO:0007569:GO:0000122:GO:0007417:GO:0043234:GO:0072332:GO:0005654:GO:0019899:GO:0045893:GO:2000379:GO:0045892:GO:0051276:GO:0007369:GO:0005678:GO:0008285:GO:0010165:GO:0008283:GO:0090403:GO:0007265:GO:0005739:GO:0002039:GO:0005737:GO:0008104:GO:0035264:GO:0045944:GO:0001085:GO:0007050:GO:0042149:GO:0030512:GO:0006355:GO:0043066:GO:0051262:GO:0005669:GO:0008635:GO:0043065:GO:0005730:GO:0006915:GO:0003677:GO:0005524:GO:0003684:GO:0016604:GO:0070215:GO:0047485:GO:0006461:GO:0003682:GO:0016605:GO:0034613:GO:0008629	191170	PF08563:PF07710:PF00870	2.36,0.13,-0.88,0.2,0.15,-0.39,-1.0,-1.96,-1.18,0.06,0.06,0.46,0.46,0.38,-0.47,0.46,0.38

346

# locus	type	ref	length	genotype	pvalue	coverage	allele_coverage	maf	gene	transcript	location	function	codon	exon	protein	coding	sift	polyphen	grantham	normalizedAlt	5000Exomes	cosmic	dbsnp	drugbank	go	omim	pfam	phylop
chr17:7579470	SNV	CGG	1	CGG/CGC	1,78E-159	209	75,134,0,0,0	0.398:0.0	TP53	NM_000546.5	exonic	missense	CGC	4	p.Pro72Arg	c.215C>G			103.0	C		43787:18569:1268332:1268331:96586:45288:46307:44256:44466:1386925:1386926:46058:46408	rs1042522:rs56275308	1-(9-ethyl-9H-carbazol-3-yl)-N-methylmethanamine	GO:0005515:GO:0030308:GO:0003700:GO:0044212:GO:0009303:GO:0050731:GO:0009792:GO:0030154:GO:0034644:GO:0044419:GO:0002326:GO:0010332:GO:0046677:GO:0031625:GO:0000060:GO:0035035:GO:0035033:GO:0046982:GO:0005507:GO:0070245:GO:0007275:GO:0001701:GO:0071479:GO:0000733:GO:0042771:GO:0006284:GO:0000075:GO:0008270:GO:0006289:GO:0090399:GO:0000739:GO:0008134:GO:0005783:GO:0051087:GO:0048147:GO:0005634:GO:0001836:GO:0090343:GO:0008340:GO:0046872:GO:0016363:GO:0006302:GO:0005829:GO:0002309:GO:0051726:GO:0051721:GO:0046902:GO:0071456:GO:0042981:GO:0051097:GO:0005626:GO:0035690:GO:0006974:GO:0031065:GO:0007596:GO:0008156:GO:0031571:GO:0000790:GO:0006983:GO:0005657:GO:0001756:GO:0002020:GO:0071158:GO:0007406:GO:0042802:GO:0043525:GO:0002360:GO:0006978:GO:0000785:GO:0006977:GO:0033077:GO:0019901:GO:0030330:GO:0007569:GO:0000122:GO:0007417:GO:0043234:GO:0072332:GO:0005654:GO:0019899:GO:0045893:GO:2000379:GO:0045892:GO:0051276:GO:0007369:GO:0005678:GO:0008285:GO:0010165:GO:0008283:GO:0090403:GO:0007265:GO:0005739:GO:0002039:GO:0005737:GO:0008104:GO:0035264:GO:0045944:GO:0001085:GO:0007050:GO:0042149:GO:0030512:GO:0006355:GO:0043066:GO:0051262:GO:0005669:GO:0008635:GO:0043065:GO:0005730:GO:0006915:GO:0003677:GO:0005524:GO:0003684:GO:0016604:GO:0070215:GO:0047485:GO:0006461:GO:0003682:GO:0016605:GO:0034613:GO:0008629	191170	PF08563:PF07710:PF00870	-1.14,-0.69,1.36
chr17:7579619	SNV	G	1	G/T	3,37E-131	491	316,175	0.073	TP53	NM_000546.5	intronic										AMAF=0.1067:EMAF=0.0524:GMAF=0.0708		rs17883323	1-(9-ethyl-9H-carbazol-3-yl)-N-methylmethanamine	GO:0005515:GO:0030308:GO:0003700:GO:0044212:GO:0009303:GO:0050731:GO:0009792:GO:0030154:GO:0034644:GO:0044419:GO:0002326:GO:0010332:GO:0046677:GO:0031625:GO:0000060:GO:0035035:GO:0035033:GO:0046982:GO:0005507:GO:0070245:GO:0007275:GO:0001701:GO:0071479:GO:0000733:GO:0042771:GO:0006284:GO:0000075:GO:0008270:GO:0006289:GO:0090399:GO:0000739:GO:0008134:GO:0005783:GO:0051087:GO:0048147:GO:0005634:GO:0001836:GO:0090343:GO:0008340:GO:0046872:GO:0016363:GO:0006302:GO:0005829:GO:0002309:GO:0051726:GO:0051721:GO:0046902:GO:0071456:GO:0042981:GO:0051097:GO:0005626:GO:0035690:GO:0006974:GO:0031065:GO:0007596:GO:0008156:GO:0031571:GO:0000790:GO:0006983:GO:0005657:GO:0001756:GO:0002020:GO:0071158:GO:0007406:GO:0042802:GO:0043525:GO:0002360:GO:0006978:GO:0000785:GO:0006977:GO:0033077:GO:0019901:GO:0030330:GO:0007569:GO:0000122:GO:0007417:GO:0043234:GO:0072332:GO:0005654:GO:0019899:GO:0045893:GO:2000379:GO:0045892:GO:0051276:GO:0007369:GO:0005678:GO:0008285:GO:0010165:GO:0008283:GO:0090403:GO:0007265:GO:0005739:GO:0002039:GO:0005737:GO:0008104:GO:0035264:GO:0045944:GO:0001085:GO:0007050:GO:0042149:GO:0030512:GO:0006355:GO:0043066:GO:0051262:GO:0005669:GO:0008635:GO:0043065:GO:0005730:GO:0006915:GO:0003677:GO:0005524:GO:0003684:GO:0016604:GO:0070215:GO:0047485:GO:0006461:GO:0003682:GO:0016605:GO:0034613:GO:0008629	191170	PF08563:PF07710:PF00870	-0.7
chr17:7579643	INDEL	CCCCCAGCCCTCCAGGT	16	CCCCCAGCCCTCCAGGT/C	1,00E+10	818	605,213	0.153	TP53	NM_000546.5	intronic										AMAF=0.3385:EMAF=0.2049:GMAF=0.2511		rs59758982:rs17878362:rs150200764:rs146534833:rs139100308:rs67112752:rs66510636:rs138066738	1-(9-ethyl-9H-carbazol-3-yl)-N-methylmethanamine	GO:0005515:GO:0030308:GO:0003700:GO:0044212:GO:0009303:GO:0050731:GO:0009792:GO:0030154:GO:0034644:GO:0044419:GO:0002326:GO:0010332:GO:0046677:GO:0031625:GO:0000060:GO:0035035:GO:0035033:GO:0046982:GO:0005507:GO:0070245:GO:0007275:GO:0001701:GO:0071479:GO:0000733:GO:0042771:GO:0006284:GO:0000075:GO:0008270:GO:0006289:GO:0090399:GO:0000739:GO:0008134:GO:0005783:GO:0051087:GO:0048147:GO:0005634:GO:0001836:GO:0090343:GO:0008340:GO:0046872:GO:0016363:GO:0006302:GO:0005829:GO:0002309:GO:0051726:GO:0051721:GO:0046902:GO:0071456:GO:0042981:GO:0051097:GO:0005626:GO:0035690:GO:0006974:GO:0031065:GO:0007596:GO:0008156:GO:0031571:GO:0000790:GO:0006983:GO:0005657:GO:0001756:GO:0002020:GO:0071158:GO:0007406:GO:0042802:GO:0043525:GO:0002360:GO:0006978:GO:0000785:GO:0006977:GO:0033077:GO:0019901:GO:0030330:GO:0007569:GO:0000122:GO:0007417:GO:0043234:GO:0072332:GO:0005654:GO:0019899:GO:0045893:GO:2000379:GO:0045892:GO:0051276:GO:0007369:GO:0005678:GO:0008285:GO:0010165:GO:0008283:GO:0090403:GO:0007265:GO:0005739:GO:0002039:GO:0005737:GO:0008104:GO:0035264:GO:0045944:GO:0001085:GO:0007050:GO:0042149:GO:0030512:GO:0006355:GO:0043066:GO:0051262:GO:0005669:GO:0008635:GO:0043065:GO:0005730:GO:0006915:GO:0003677:GO:0005524:GO:0003684:GO:0016604:GO:0070215:GO:0047485:GO:0006461:GO:0003682:GO:0016605:GO:0034613:GO:0008629	191170	PF08563:PF07710:PF00870	2.36,0.13,-0.88,0.2,0.15,-0.39,-1.0,-1.96,-1.18,0.06,0.06,0.46,0.46,0.38,-0.47,0.46,0.38

521

SIFT	PolyPhen2	LRT	MutationTaster	MutationAssessor	FATHMM	MetaSVM	Occurrences	HGMD	PROVEAN	# locus	type	ref	length	genotype	pvalue	coverage	allele_coverage	maf	gene	transcript	location	function	codon	exon	protein	coding	sift	polyphen	grantham	normalizedAlt	5000Exomes	cosmic	dbsnp	drugbank	go	omim	pfam	phylop
-	-	-	-	-	-	-	0	17 7579470 3327 CGG CGC	17,7579470,CGG,CGC	chr17:7579470	SNV	CGG	1	CGG/CGC	0.0	408	7,401,0,0,0	0.398:0.0	TP53	NM_000546.5	exonic	missense	CGC	4	p.Pro72Arg	c.215C>G			103.0	C		43787:18569:1268332:1268331:96586:45288:46307:44256:44466:1386925:1386926:46058:46408	rs1042522:rs56275308	1-(9-ethyl-9H-carbazol-3-yl)-N-methylmethanamine	GO:0005515:GO:0030308:GO:0003700:GO:0044212:GO:0009303:GO:0050731:GO:0009792:GO:0030154:GO:0034644:GO:0044419:GO:0002326:GO:0010332:GO:0046677:GO:0031625:GO:0000060:GO:0035035:GO:0035033:GO:0046982:GO:0005507:GO:0070245:GO:0007275:GO:0001701:GO:0071479:GO:0000733:GO:0042771:GO:0006284:GO:0000075:GO:0008270:GO:0006289:GO:0090399:GO:0000739:GO:0008134:GO:0005783:GO:0051087:GO:0048147:GO:0005634:GO:0001836:GO:0090343:GO:0008340:GO:0046872:GO:0016363:GO:0006302:GO:0005829:GO:0002309:GO:0051726:GO:0051721:GO:0046902:GO:0071456:GO:0042981:GO:0051097:GO:0005626:GO:0035690:GO:0006974:GO:0031065:GO:0007596:GO:0008156:GO:0031571:GO:0000790:GO:0006983:GO:0005657:GO:0001756:GO:0002020:GO:0071158:GO:0007406:GO:0042802:GO:0043525:GO:0002360:GO:0006978:GO:0000785:GO:0006977:GO:0033077:GO:0019901:GO:0030330:GO:0007569:GO:0000122:GO:0007417:GO:0043234:GO:0072332:GO:0005654:GO:0019899:GO:0045893:GO:2000379:GO:0045892:GO:0051276:GO:0007369:GO:0005678:GO:0008285:GO:0010165:GO:0008283:GO:0090403:GO:0007265:GO:0005739:GO:0002039:GO:0005737:GO:0008104:GO:0035264:GO:0045944:GO:0001085:GO:0007050:GO:0042149:GO:0030512:GO:0006355:GO:0043066:GO:0051262:GO:0005669:GO:0008635:GO:0043065:GO:0005730:GO:0006915:GO:0003677:GO:0005524:GO:0003684:GO:0016604:GO:0070215:GO:0047485:GO:0006461:GO:0003682:GO:0016605:GO:0034613:GO:0008629	191170	PF08563:PF07710:PF00870	-1.14,-0.69,1.36

-	-	-	-	-	-	-	12	17 7579643 3356 CCCCCAGCCCTCCAGGT C	17,7579643,CCCCCAGCCCTCCAGGT,C	chr17:7579643	INDEL	CCCCCAGCCCTCCAGGT	16	CCCCCAGCCCTCCAGGT/C	1,00E+10	530	345,185	0.153	TP53	NM_000546.5	intronic										AMAF=0.3385:EMAF=0.2049:GMAF=0.2511		rs59758982:rs17878362:rs150200764:rs146534833:rs139100308:rs67112752:rs66510636:rs138066738	1-(9-ethyl-9H-carbazol-3-yl)-N-methylmethanamine	GO:0005515:GO:0030308:GO:0003700:GO:0044212:GO:0009303:GO:0050731:GO:0009792:GO:0030154:GO:0034644:GO:0044419:GO:0002326:GO:0010332:GO:0046677:GO:0031625:GO:0000060:GO:0035035:GO:0035033:GO:0046982:GO:0005507:GO:0070245:GO:0007275:GO:0001701:GO:0071479:GO:0000733:GO:0042771:GO:0006284:GO:0000075:GO:0008270:GO:0006289:GO:0090399:GO:0000739:GO:0008134:GO:0005783:GO:0051087:GO:0048147:GO:0005634:GO:0001836:GO:0090343:GO:0008340:GO:0046872:GO:0016363:GO:0006302:GO:0005829:GO:0002309:GO:0051726:GO:0051721:GO:0046902:GO:0071456:GO:0042981:GO:0051097:GO:0005626:GO:0035690:GO:0006974:GO:0031065:GO:0007596:GO:0008156:GO:0031571:GO:0000790:GO:0006983:GO:0005657:GO:0001756:GO:0002020:GO:0071158:GO:0007406:GO:0042802:GO:0043525:GO:0002360:GO:0006978:GO:0000785:GO:0006977:GO:0033077:GO:0019901:GO:0030330:GO:0007569:GO:0000122:GO:0007417:GO:0043234:GO:0072332:GO:0005654:GO:0019899:GO:0045893:GO:2000379:GO:0045892:GO:0051276:GO:0007369:GO:0005678:GO:0008285:GO:0010165:GO:0008283:GO:0090403:GO:0007265:GO:0005739:GO:0002039:GO:0005737:GO:0008104:GO:0035264:GO:0045944:GO:0001085:GO:0007050:GO:0042149:GO:0030512:GO:0006355:GO:0043066:GO:0051262:GO:0005669:GO:0008635:GO:0043065:GO:0005730:GO:0006915:GO:0003677:GO:0005524:GO:0003684:GO:0016604:GO:0070215:GO:0047485:GO:0006461:GO:0003682:GO:0016605:GO:0034613:GO:0008629	191170	PF08563:PF07710:PF00870	2.36,0.13,-0.88,0.2,0.15,-0.39,-1.0,-1.96,-1.18,0.06,0.06,0.46,0.46,0.38,-0.47,0.46,0.38

840

SIFT	PolyPhen2	LRT	MutationTaster	MutationAssessor	FATHMM	MetaSVM	Occurrences	HGMD	PROVEAN	# locus	type	ref	length	genotype	pvalue	coverage	allele_coverage	maf	gene	transcript	location	function	codon	exon	protein	coding	sift	polyphen	grantham	normalizedAlt	5000Exomes	cosmic	dbsnp	drugbank	go	omim	pfam	phylop
-	-	-	-	-	-	-	0	17 7579470 3547 CGG CGC	17,7579470,CGG,CGC	chr17:7579470	SNV	CGG	1	CGC/CGC	0.0	1424	0,1424,0,0,0	0.398:0.0	TP53	NM_000546.5	exonic	missense	CGC	4	p.Pro72Arg	c.215C>G			103.0	C		43787:18569:1268332:1268331:96586:45288:46307:44256:44466:1386925:1386926:46058:46408	rs1042522:rs56275308	1-(9-ethyl-9H-carbazol-3-yl)-N-methylmethanamine	GO:0005515:GO:0030308:GO:0003700:GO:0044212:GO:0009303:GO:0050731:GO:0009792:GO:0030154:GO:0034644:GO:0044419:GO:0002326:GO:0010332:GO:0046677:GO:0031625:GO:0000060:GO:0035035:GO:0035033:GO:0046982:GO:0005507:GO:0070245:GO:0007275:GO:0001701:GO:0071479:GO:0000733:GO:0042771:GO:0006284:GO:0000075:GO:0008270:GO:0006289:GO:0090399:GO:0000739:GO:0008134:GO:0005783:GO:0051087:GO:0048147:GO:0005634:GO:0001836:GO:0090343:GO:0008340:GO:0046872:GO:0016363:GO:0006302:GO:0005829:GO:0002309:GO:0051726:GO:0051721:GO:0046902:GO:0071456:GO:0042981:GO:0051097:GO:0005626:GO:0035690:GO:0006974:GO:0031065:GO:0007596:GO:0008156:GO:0031571:GO:0000790:GO:0006983:GO:0005657:GO:0001756:GO:0002020:GO:0071158:GO:0007406:GO:0042802:GO:0043525:GO:0002360:GO:0006978:GO:0000785:GO:0006977:GO:0033077:GO:0019901:GO:0030330:GO:0007569:GO:0000122:GO:0007417:GO:0043234:GO:0072332:GO:0005654:GO:0019899:GO:0045893:GO:2000379:GO:0045892:GO:0051276:GO:0007369:GO:0005678:GO:0008285:GO:0010165:GO:0008283:GO:0090403:GO:0007265:GO:0005739:GO:0002039:GO:0005737:GO:0008104:GO:0035264:GO:0045944:GO:0001085:GO:0007050:GO:0042149:GO:0030512:GO:0006355:GO:0043066:GO:0051262:GO:0005669:GO:0008635:GO:0043065:GO:0005730:GO:0006915:GO:0003677:GO:0005524:GO:0003684:GO:0016604:GO:0070215:GO:0047485:GO:0006461:GO:0003682:GO:0016605:GO:0034613:GO:0008629	191170	PF08563:PF07710:PF00870	-1.14,-0.69,1.36
-	-	-	-	-	-	-	12	17 7579643 3576 CCCCCAGCCCTCCAGGT C	17,7579643,CCCCCAGCCCTCCAGGT,C	chr17:7579643	INDEL	CCCCCAGCCCTCCAGGT	16	CCCCCAGCCCTCCAGGT/C	1,00E+10	2536	1831,705	0.153	TP53	NM_000546.5	intronic										AMAF=0.3385:EMAF=0.2049:GMAF=0.2511		rs59758982:rs17878362:rs150200764:rs146534833:rs139100308:rs67112752:rs66510636:rs138066738	1-(9-ethyl-9H-carbazol-3-yl)-N-methylmethanamine	GO:0005515:GO:0030308:GO:0003700:GO:0044212:GO:0009303:GO:0050731:GO:0009792:GO:0030154:GO:0034644:GO:0044419:GO:0002326:GO:0010332:GO:0046677:GO:0031625:GO:0000060:GO:0035035:GO:0035033:GO:0046982:GO:0005507:GO:0070245:GO:0007275:GO:0001701:GO:0071479:GO:0000733:GO:0042771:GO:0006284:GO:0000075:GO:0008270:GO:0006289:GO:0090399:GO:0000739:GO:0008134:GO:0005783:GO:0051087:GO:0048147:GO:0005634:GO:0001836:GO:0090343:GO:0008340:GO:0046872:GO:0016363:GO:0006302:GO:0005829:GO:0002309:GO:0051726:GO:0051721:GO:0046902:GO:0071456:GO:0042981:GO:0051097:GO:0005626:GO:0035690:GO:0006974:GO:0031065:GO:0007596:GO:0008156:GO:0031571:GO:0000790:GO:0006983:GO:0005657:GO:0001756:GO:0002020:GO:0071158:GO:0007406:GO:0042802:GO:0043525:GO:0002360:GO:0006978:GO:0000785:GO:0006977:GO:0033077:GO:0019901:GO:0030330:GO:0007569:GO:0000122:GO:0007417:GO:0043234:GO:0072332:GO:0005654:GO:0019899:GO:0045893:GO:2000379:GO:0045892:GO:0051276:GO:0007369:GO:0005678:GO:0008285:GO:0010165:GO:0008283:GO:0090403:GO:0007265:GO:0005739:GO:0002039:GO:0005737:GO:0008104:GO:0035264:GO:0045944:GO:0001085:GO:0007050:GO:0042149:GO:0030512:GO:0006355:GO:0043066:GO:0051262:GO:0005669:GO:0008635:GO:0043065:GO:0005730:GO:0006915:GO:0003677:GO:0005524:GO:0003684:GO:0016604:GO:0070215:GO:0047485:GO:0006461:GO:0003682:GO:0016605:GO:0034613:GO:0008629	191170	PF08563:PF07710:PF00870	2.36,0.13,-0.88,0.2,0.15,-0.39,-1.0,-1.96,-1.18,0.06,0.06,0.46,0.46,0.38,-0.47,0.46,0.38

829

SIFT	PolyPhen2	LRT	MutationTaster	MutationAssessor	FATHMM	MetaSVM	Occurrences	HGMD	PROVEAN	# locus	type	ref	length	genotype	pvalue	coverage	allele_coverage	maf	gene	transcript	location	function	codon	exon	protein	coding	sift	polyphen	grantham	normalizedAlt	5000Exomes	cosmic	dbsnp	drugbank	go	omim	pfam	phylop
.	P	.	D	.	D	D	0	17 7576645 3008 A G	17,7576645,A,G	chr17:7576645	SNV	A	1	A/G	0.16143585568264862	56	54,2		TP53	NM_000546.5	intronic													1-(9-ethyl-9H-carbazol-3-yl)-N-methylmethanamine	GO:0005515:GO:0030308:GO:0003700:GO:0044212:GO:0009303:GO:0050731:GO:0009792:GO:0030154:GO:0034644:GO:0044419:GO:0002326:GO:0010332:GO:0046677:GO:0031625:GO:0000060:GO:0035035:GO:0035033:GO:0046982:GO:0005507:GO:0070245:GO:0007275:GO:0001701:GO:0071479:GO:0000733:GO:0042771:GO:0006284:GO:0000075:GO:0008270:GO:0006289:GO:0090399:GO:0000739:GO:0008134:GO:0005783:GO:0051087:GO:0048147:GO:0005634:GO:0001836:GO:0090343:GO:0008340:GO:0046872:GO:0016363:GO:0006302:GO:0005829:GO:0002309:GO:0051726:GO:0051721:GO:0046902:GO:0071456:GO:0042981:GO:0051097:GO:0005626:GO:0035690:GO:0006974:GO:0031065:GO:0007596:GO:0008156:GO:0031571:GO:0000790:GO:0006983:GO:0005657:GO:0001756:GO:0002020:GO:0071158:GO:0007406:GO:0042802:GO:0043525:GO:0002360:GO:0006978:GO:0000785:GO:0006977:GO:0033077:GO:0019901:GO:0030330:GO:0007569:GO:0000122:GO:0007417:GO:0043234:GO:0072332:GO:0005654:GO:0019899:GO:0045893:GO:2000379:GO:0045892:GO:0051276:GO:0007369:GO:0005678:GO:0008285:GO:0010165:GO:0008283:GO:0090403:GO:0007265:GO:0005739:GO:0002039:GO:0005737:GO:0008104:GO:0035264:GO:0045944:GO:0001085:GO:0007050:GO:0042149:GO:0030512:GO:0006355:GO:0043066:GO:0051262:GO:0005669:GO:0008635:GO:0043065:GO:0005730:GO:0006915:GO:0003677:GO:0005524:GO:0003684:GO:0016604:GO:0070215:GO:0047485:GO:0006461:GO:0003682:GO:0016605:GO:0034613:GO:0008629	191170	PF08563:PF07710:PF00870	1.08
-	-	-	-	-	-	-	0	17 7579470 3544 CGG CGC	17,7579470,CGG,CGC	chr17:7579470	SNV	CGG	1	CGG/CGC	4.073803E-317	453	203,249,0,1,0	0.398:0.0	TP53	NM_000546.5	exonic	missense	CGC	4	p.Pro72Arg	c.215C>G			103.0	C		43787:18569:1268332:1268331:96586:45288:46307:44256:44466:1386925:1386926:46058:46408	rs1042522:rs56275308	1-(9-ethyl-9H-carbazol-3-yl)-N-methylmethanamine	GO:0005515:GO:0030308:GO:0003700:GO:0044212:GO:0009303:GO:0050731:GO:0009792:GO:0030154:GO:0034644:GO:0044419:GO:0002326:GO:0010332:GO:0046677:GO:0031625:GO:0000060:GO:0035035:GO:0035033:GO:0046982:GO:0005507:GO:0070245:GO:0007275:GO:0001701:GO:0071479:GO:0000733:GO:0042771:GO:0006284:GO:0000075:GO:0008270:GO:0006289:GO:0090399:GO:0000739:GO:0008134:GO:0005783:GO:0051087:GO:0048147:GO:0005634:GO:0001836:GO:0090343:GO:0008340:GO:0046872:GO:0016363:GO:0006302:GO:0005829:GO:0002309:GO:0051726:GO:0051721:GO:0046902:GO:0071456:GO:0042981:GO:0051097:GO:0005626:GO:0035690:GO:0006974:GO:0031065:GO:0007596:GO:0008156:GO:0031571:GO:0000790:GO:0006983:GO:0005657:GO:0001756:GO:0002020:GO:0071158:GO:0007406:GO:0042802:GO:0043525:GO:0002360:GO:0006978:GO:0000785:GO:0006977:GO:0033077:GO:0019901:GO:0030330:GO:0007569:GO:0000122:GO:0007417:GO:0043234:GO:0072332:GO:0005654:GO:0019899:GO:0045893:GO:2000379:GO:0045892:GO:0051276:GO:0007369:GO:0005678:GO:0008285:GO:0010165:GO:0008283:GO:0090403:GO:0007265:GO:0005739:GO:0002039:GO:0005737:GO:0008104:GO:0035264:GO:0045944:GO:0001085:GO:0007050:GO:0042149:GO:0030512:GO:0006355:GO:0043066:GO:0051262:GO:0005669:GO:0008635:GO:0043065:GO:0005730:GO:0006915:GO:0003677:GO:0005524:GO:0003684:GO:0016604:GO:0070215:GO:0047485:GO:0006461:GO:0003682:GO:0016605:GO:0034613:GO:0008629	191170	PF08563:PF07710:PF00870	-1.14,-0.69,1.36
