# Supplementary material for: RNA-Seq reveals the existence of a CDKN1C-E2F1-TP53 axis that is altered in human T-cell lymphoblastic lymphomas
Source: BMC Cancer. 2018 Apr 16;18:430. doi: 10.1186/s12885-018-4304-y (PMC5902834; doi:10.1186/s12885-018-4304-y)
Supplement: Supplementary file 8 — Table S6. MicroRNA regulation of CDKN1C, E2F1 and TP53 genes in T-TLBLs of the exploratory cohort by RNA-Seq. (PDF 78 kb) [file 12885_2018_4304_MOESM8_ESM.pdf]

Supplementary Table 6. MicroRNA regulation of CDKN1C, E2F1 and TP53 genes in T-TLBLs of the exploratory cohort by RNA-Seq.

miRNA	Gene target	Sample								
		control	840	460	521	238	192	346	408	554
hsa-miR-221-3p	CDKN1C	1600,02	17271,02	3400,21	2753,92	4469,38	2502,02	2895,39	3757,41	1870,87
hsa-miR-222-3p	CDKN1C	1574,66	9816,38	1666,03	2801,27	4123,51	3153,06	1265,65	2065,70	1301,16
hsa-miR-17-5p	E2F1	1761,14	9076,96	1366,54	2134,54	2517,03	1581,99	1937,49	4565,21	4401,98
hsa-miR-203a	E2F1	873,42	61,06	193,68	10,24	2,52	7,02	17,34	6,04	9,16
hsa-miR-205-5p	E2F1	15153,48	442,67	48,42	3,84	9,26	8,58	10,84	6,65	12,21
hsa-miR-21-5p	E2F1	19124,87	87901,11	52319,42	48929,43	104336,68	60352,50	38077,88	82151,95	68850,72
hsa-miR-223-3p	E2F1	589,19	8004,17	5371,11	854,84	700,16	379,71	1165,96	1749,11	507,65
hsa-miR-20a-5p	E2F1	3981,82	11721,41	3507,81	5249,34	2974,82	3081,33	5056,10	3728,41	3932,99
hsa-miR-331-3p	E2F1	183,01	501,90	242,10	168,92	284,44	222,21	134,37	431,99	379,46
hsa-miR-10b-5p	TP53	44169,67	64108,43	95854,97	62190,99	156031,24	63471,25	55363,54	181389,58	119943,99
hsa-miR-125b-5p	TP53	3857,72	1020,29	898,47	836,93	1813,50	1067,39	981,75	1074,24	1030,55
hsa-miR-150-5p	TP53	12949,38	3587,19	33399,49	39485,22	33292,75	6525,21	23388,53	14230,92	2063,14
hsa-miR-200a-3p	TP53	1181,61	68,39	8,97	8,96	12,62	7,02	15,17	3,63	2,03
hsa-miR-30e-5p	TP53	32594,02	46065,62	16289,07	22387,12	5830,14	23312,66	39709,78	7720,85	8103,02
hsa-miR-375	TP53	672,95	79,99	41,25	5,12	14,31	5,46	10,84	64,04	0,00
hsa-miR-19b-3p	TP53	5682,11	21135,42	5772,82	6178,40	3916,50	4298,42	6551,48	6663,53	4720,40
hsa-miR-214-3p	TP53	180,56	52,51	127,33	97,26	197,76	222,21	140,87	238,65	156,67
hsa-let-7a-3p	TP53	98,13	249,73	236,72	268,74	128,75	293,94	312,08	346,80	283,83
hsa-let-7b-3p	TP53	18,14	50,68	285,14	227,79	120,34	252,62	212,39	314,18	260,44
hsa-let-7f-1-3p	TP53	19,23	7,94	69,94	117,73	56,38	132,55	93,19	91,84	120,04
hsa-let-7b-5p	TP53	1169,81	2389,22	8798,21	5399,07	5137,56	6051,94	6601,32	10352,07	7025,67
hsa-let-7d-5p	TP53	1370,14	768,73	2756,39	2794,87	3385,49	2473,95	2828,21	2420,96	3223,91
hsa-let-7f-5p	TP53	31321,99	32829,96	93654,52	95763,99	116804,84	67506,92	66149,74	67698,08	52563,30
hsa-let-7g-5p	TP53	11292,38	10671,20	29995,70	29832,43	41703,03	18002,21	25570,91	27950,71	17854,11
hsa-let-7i-5p	TP53	7199,97	8728,93	13338,99	15617,49	19046,43	14907,63	13154,96	22120,95	11302,51
hsa-let-7c-5p	TP53	4451,18	1404,35	853,64	793,42	1436,50	328,25	1083,60	759,46	1438,50

Read counts of deregulated miRNA by gene in each sample from the exploratory cohort are shown.
