# Supplementary material for: RNA-Seq reveals the existence of a CDKN1C-E2F1-TP53 axis that is altered in human T-cell lymphoblastic lymphomas
Source: BMC Cancer. 2018 Apr 16;18:430. doi: 10.1186/s12885-018-4304-y (PMC5902834; doi:10.1186/s12885-018-4304-y)
Supplement: Supplementary file 9 — Table S7. Validation analysis by qRT-PCR of those miRNA significantly deregulated according with RNA-Seq analysis in the exploratory and extended cohort of T-LBL samples. (PDF 81 kb) [file 12885_2018_4304_MOESM9_ESM.pdf]

**Supplementary Table 7. Validation analysis by qRT-PCR of those miRNA significantly deregulated according with RNA-Seq analysis in the exploratory and extended cohort of T-LBL samples.**

| Sample Cohort | Sample | miRNA_id | gene symbol target | Housekeeping | 2^-DCt 2_1_control | 2^-DCt 2_2_tumor | P value | Norm_1 | Norm_2 | log2FC |
|---|---|---|---|---|---|---|---|---|---|---|
| Exploratory cohort | 554 | hsa-miR-200a-3p | TP53 | miR-SNORD48 | 147,380 | 0,080 | 0,000 | 1,000 | 0,001 | -10,970 |
| | | hsa-miR-203a | E2F1 | miR-SNORD48 | 223,320 | 53,490 | 0,017 | 1,000 | 0,239 | -2,064 |
| | | hsa-miR-205-5p | E2F1 | miR-SNORD48 | 311,740 | 1,920 | 0,001 | 1,000 | 0,006 | -7,356 |
| | | hsa-miR-221-3p | CDKN1C | miR-SNORD48 | 1,070 | 1,410 | 0,853 | 1,000 | 1,317 | 0,397 |
| | | hsa-miR-222-3p | CDKN1C | miR-SNORD48 | 1,390 | 0,180 | 0,022 | 1,000 | 0,129 | -2,954 |
| | | hsa-miR-375 | TP53 | miR-SNORD48 | 3,460 | 17,240 | 0,021 | 1,000 | 4,982 | 2,317 |
| | 238 | hsa-miR-200a-3p | TP53 | miR-SNORD48 | 147,380 | 0,750 | 0,000 | 1,000 | 0,005 | -6,643 |
| | | hsa-miR-203a | E2F1 | miR-SNORD48 | 223,320 | 0,370 | 0,016 | 1,000 | 0,002 | -9,287 |
| | | hsa-miR-205-5p | E2F1 | miR-SNORD48 | 311,740 | 0,790 | 0,011 | 1,000 | 0,003 | -8,643 |
| | | hsa-miR-221-3p | CDKN1C | miR-SNORD48 | 1,070 | 1,180 | 0,613 | 1,000 | 1,103 | 0,028 |
| | | hsa-miR-222-3p | CDKN1C | miR-SNORD48 | 1,390 | 2,450 | 0,181 | 1,000 | 1,763 | 0,817 |
| | | hsa-miR-375 | TP53 | miR-SNORD48 | 3,460 | 0,820 | 0,001 | 1,000 | 0,237 | -2,077 |
| | 408 | hsa-miR-200a-3p | TP53 | miR-SNORD48 | 147,380 | 0,600 | 0,000 | 1,000 | 0,004 | -7,930 |
| | | hsa-miR-203a | E2F1 | miR-SNORD48 | 223,320 | 0,960 | 0,016 | 1,000 | 0,004 | -7,895 |
| | | hsa-miR-205-5p | E2F1 | miR-SNORD48 | 311,740 | 1,850 | 0,011 | 1,000 | 0,006 | -7,405 |
| | | hsa-miR-221-3p | CDKN1C | miR-SNORD48 | 1,070 | 0,430 | 0,066 | 1,000 | 0,402 | -1,315 |
| | | hsa-miR-222-3p | CDKN1C | miR-SNORD48 | 1,390 | 1,280 | 0,455 | 1,000 | 0,921 | -0,119 |
| | | hsa-miR-375 | TP53 | miR-SNORD48 | 3,460 | 1,250 | 0,003 | 1,000 | 0,361 | -1,469 |
| | 192 | hsa-miR-200a-3p | TP53 | miR-SNORD48 | 147,380 | 0,760 | 0,000 | 1,000 | 0,005 | -7,615 |
| | | hsa-miR-203a | E2F1 | miR-SNORD48 | 223,320 | 0,610 | 0,016 | 1,000 | 0,003 | -8,533 |
| | | hsa-miR-205-5p | E2F1 | miR-SNORD48 | 311,740 | 0,100 | 0,011 | 1,000 | 0,000 | -11,700 |
| | | hsa-miR-221-3p | CDKN1C | miR-SNORD48 | 1,070 | 0,720 | 0,011 | 1,000 | 0,518 | -0,949 |
| | | hsa-miR-222-3p | CDKN1C | miR-SNORD48 | 1,390 | 0,440 | 0,012 | 1,000 | 0,316 | -1,662 |
| | | hsa-miR-375 | TP53 | miR-SNORD48 | 3,460 | 1,110 | 0,022 | 1,000 | 0,321 | -1,639 |
| | 460 | hsa-miR-200a-3p | TP53 | miR-SNORD48 | 147,380 | 0,000 | 0,000 | 1,000 | 0,000 | -22,250 |
| | | hsa-miR-203a | E2F1 | miR-SNORD48 | 223,320 | 3,110 | 0,017 | 1,000 | 0,014 | -6,158 |
| | | hsa-miR-205-5p | E2F1 | miR-SNORD48 | 311,740 | 0,880 | 0,011 | 1,000 | 0,003 | -8,381 |
| | | hsa-miR-221-3p | CDKN1C | miR-SNORD48 | 1,070 | 0,390 | 0,019 | 1,000 | 0,364 | -1,458 |
| | | hsa-miR-222-3p | CDKN1C | miR-SNORD48 | 1,390 | 0,730 | 0,036 | 1,000 | 0,525 | -0,929 |
| | | hsa-miR-375 | TP53 | miR-SNORD48 | 3,460 | 0,450 | 0,024 | 1,000 | 0,130 | -2,943 |
| | 346 | hsa-miR-200a-3p | TP53 | miR-SNORD48 | 147,380 | 0,610 | 0,000 | 1,000 | 0,004 | -7,930 |
| | | hsa-miR-203a | E2F1 | miR-SNORD48 | 223,320 | 0,690 | 0,016 | 1,000 | 0,003 | -8,333 |
| | | hsa-miR-205-5p | E2F1 | miR-SNORD48 | 311,740 | 6,920 | 0,011 | 1,000 | 0,022 | -5,506 |
| | | hsa-miR-221-3p | CDKN1C | miR-SNORD48 | 1,070 | 1,230 | 0,713 | 1,000 | 1,149 | 0,200 |
| | | hsa-miR-222-3p | CDKN1C | miR-SNORD48 | 1,390 | 1,430 | 0,681 | 1,000 | 1,029 | 0,041 |
| | | hsa-miR-375 | TP53 | miR-SNORD48 | 3,460 | 2,010 | 0,044 | 1,000 | 0,581 | -0,783 |
| | 840 | hsa-miR-200a-3p | TP53 | miR-SNORD48 | 147,380 | 0,130 | 0,000 | 1,000 | 0,001 | -10,120 |
| | | hsa-miR-203a | E2F1 | miR-SNORD48 | 223,320 | 0,070 | 0,016 | 1,000 | 0,000 | -11,700 |
| | | hsa-miR-205-5p | E2F1 | miR-SNORD48 | 311,740 | 0,330 | 0,011 | 1,000 | 0,001 | -9,828 |
| | | hsa-miR-221-3p | CDKN1C | miR-SNORD48 | 1,070 | 2,010 | 0,366 | 1,000 | 1,878 | 0,909 |
| | | hsa-miR-222-3p | CDKN1C | miR-SNORD48 | 1,390 | 1,230 | 0,541 | 1,000 | 0,884 | -0,179 |
| | | hsa-miR-375 | TP53 | miR-SNORD48 | 3,460 | 0,250 | 0,005 | 1,000 | 0,072 | -3,795 |
| | 521 | hsa-miR-200a-3p | TP53 | miR-SNORD48 | 147,380 | 1,044 | 0,000 | 1,000 | 0,007 | -7,158 |
| | | hsa-miR-203a | E2F1 | miR-SNORD48 | 223,320 | 1,280 | 0,016 | 1,000 | 0,006 | -7,381 |
| | | hsa-miR-205-5p | E2F1 | miR-SNORD48 | 311,740 | 0,529 | 0,011 | 1,000 | 0,002 | -8,965 |
| | | hsa-miR-221-3p | CDKN1C | miR-SNORD48 | 1,070 | 1,040 | 0,593 | 1,000 | 0,972 | -0,041 |
| | | hsa-miR-222-3p | CDKN1C | miR-SNORD48 | 1,390 | 0,990 | 0,098 | 1,000 | 0,712 | -0,490 |
| | | hsa-miR-375 | TP53 | miR-SNORD48 | 3,460 | 0,405 | 0,003 | 1,000 | 0,118 | -3,083 |
| Extended cohort | 829 | hsa-miR-200a-3p | TP53 | miR-SNORD48 | 147,380 | 0,820 | 0,000 | 1,000 | 0,006 | -7,506 |
| | | hsa-miR-203a | E2F1 | miR-SNORD48 | 223,320 | 0,760 | 0,016 | 1,000 | 0,003 | -8,200 |
| | | hsa-miR-205-5p | E2F1 | miR-SNORD48 | 311,740 | 0,350 | 0,011 | 1,000 | 0,001 | -9,828 |
| | | hsa-miR-221-3p | CDKN1C | miR-SNORD48 | 1,070 | 3,430 | 0,006 | 1,000 | 3,205 | 1,680 |
| | | hsa-miR-222-3p | CDKN1C | miR-SNORD48 | 1,390 | 2,580 | 0,177 | 1,000 | 1,856 | 0,892 |
| | | hsa-miR-375 | TP53 | miR-SNORD48 | 3,460 | 0,600 | 0,003 | 1,000 | 0,173 | -2,531 |
| | 188 | hsa-miR-200a-3p | TP53 | miR-SNORD48 | 147,380 | 0,990 | 0,000 | 1,000 | 0,007 | -7,221 |
| | | hsa-miR-203a | E2F1 | miR-SNORD48 | 223,320 | 0,640 | 0,016 | 1,000 | 0,003 | -8,480 |
| | | hsa-miR-205-5p | E2F1 | miR-SNORD48 | 311,740 | 1,160 | 0,011 | 1,000 | 0,004 | -8,078 |
| | | hsa-miR-221-3p | CDKN1C | miR-SNORD48 | 1,070 | 1,250 | 0,803 | 1,000 | 1,168 | 0,224 |
| | | hsa-miR-222-3p | CDKN1C | miR-SNORD48 | 1,390 | 0,800 | 2,811 | 1,000 | 0,575 | -0,798 |
| | | hsa-miR-375 | TP53 | miR-SNORD48 | 3,460 | 0,530 | 0,022 | 1,000 | 0,153 | -2,708 |
| | 080 | hsa-miR-200a-3p | TP53 | miR-SNORD48 | 147,380 | 0,370 | 0,000 | 1,000 | 0,003 | -8,643 |
| | | hsa-miR-203a | E2F1 | miR-SNORD48 | 223,320 | 0,520 | 0,016 | 1,000 | 0,002 | -8764,000 |
| | | hsa-miR-205-5p | E2F1 | miR-SNORD48 | 311,740 | 1,030 | 0,011 | 1,000 | 0,003 | -8,243 |
| | | hsa-miR-221-3p | CDKN1C | miR-SNORD48 | 1,070 | 0,660 | 0,124 | 1,000 | 0,616 | -0,698 |
| | | hsa-miR-222-3p | CDKN1C | miR-SNORD48 | 1,390 | 3,790 | 0,055 | 1,000 | 2,726 | 1,446 |
| | | hsa-miR-375 | TP53 | miR-SNORD48 | 3,460 | 0,430 | 0,001 | 1,000 | 0,124 | -3011,000 |
| | 135 | hsa-miR-200a-3p | TP53 | miR-SNORD48 | 147,380 | 0,100 | 0,000 | 1,000 | 0,001 | -10,480 |
| | | hsa-miR-203a | E2F1 | miR-SNORD48 | 223,320 | 0,080 | 0,016 | 1,000 | 0,000 | -11,287 |
| | | hsa-miR-205-5p | E2F1 | miR-SNORD48 | 311,740 | 1,010 | 0,011 | 1,000 | 0,003 | -8,287 |
| | | hsa-miR-221-3p | CDKN1C | miR-SNORD48 | 1,070 | 0,170 | 0,046 | 1,000 | 0,158 | -2,662 |
| | | hsa-miR-222-3p | CDKN1C | miR-SNORD48 | 1,390 | 0,300 | 0,016 | 1,000 | 0,215 | -2,217 |
| | | hsa-miR-375 | TP53 | miR-SNORD48 | 3,460 | 1,620 | 0,011 | 1,000 | 0,468 | -1,095 |
| | 104 | hsa-miR-200a-3p | TP53 | miR-SNORD48 | 147,380 | 0,290 | 0,000 | 1,000 | 0,002 | -9,039 |
| | | hsa-miR-203a | E2F1 | miR-SNORD48 | 223,320 | 0,470 | 0,016 | 1,000 | 0,002 | -8,895 |
| | | hsa-miR-205-5p | E2F1 | miR-SNORD48 | 311,740 | 0,720 | 0,011 | 1,000 | 0,002 | -8,965 |
| | | hsa-miR-221-3p | CDKN1C | miR-SNORD48 | 1,070 | 0,630 | 0,122 | 1,000 | 0,588 | -0,766 |
| | | hsa-miR-222-3p | CDKN1C | miR-SNORD48 | 1,390 | 0,690 | 0,073 | 1,000 | 0,496 | -1,011 |
| | | hsa-miR-375 | TP53 | miR-SNORD48 | 3,460 | 1,540 | 0,079 | 1,000 | 0,445 | -1,168 |
| | 038 | hsa-miR-200a-3p | TP53 | miR-SNORD48 | 147,380 | 92,570 | 0,024 | 1,000 | 0,628 | -0,671 |
| | | hsa-miR-203a | E2F1 | miR-SNORD48 | 223,320 | 67,330 | 0,028 | 1,000 | 0,301 | -1,732 |
| | | hsa-miR-205-5p | E2F1 | miR-SNORD48 | 311,740 | 87,720 | 0,025 | 1,000 | 0,281 | -1,831 |
| | | hsa-miR-221-3p | CDKN1C | miR-SNORD48 | 1,070 | 0,460 | 0,078 | 1,000 | 0,429 | -1,221 |
| | | hsa-miR-222-3p | CDKN1C | miR-SNORD48 | 1,390 | 1,250 | 0,437 | 1,000 | 0,899 | -0,153 |
| | | hsa-miR-375 | TP53 | miR-SNORD48 | 3,460 | 6,310 | 0,030 | 1,000 | 1,823 | 0,866 |
| | 101 | hsa-miR-200a-3p | TP53 | miR-SNORD48 | 147,380 | 0,060 | 0,000 | 1,000 | 0,000 | -11,287 |
| | | hsa-miR-203a | E2F1 | miR-SNORD48 | 223,320 | 0,050 | 0,016 | 1,000 | 0,000 | -12,287 |
| | | hsa-miR-205-5p | E2F1 | miR-SNORD48 | 311,740 | 0,003 | 0,011 | 1,000 | 0,000 | -16,668 |
| | | hsa-miR-221-3p | CDKN1C | miR-SNORD48 | 1,070 | 0,740 | 0,160 | 1,000 | 0,691 | -0,533 |
| | | hsa-miR-222-3p | CDKN1C | miR-SNORD48 | 1,390 | 1,420 | 0,949 | 1,000 | 1,021 | 0,029 |
| | | hsa-miR-375 | TP53 | miR-SNORD48 | 3,460 | 0,230 | 0,006 | 1,000 | 0,066 | -3921,000 |
| | 209 | hsa-miR-200a-3p | TP53 | miR-SNORD48 | 147,380 | 0,510 | 0,000 | 1,000 | 0,003 | -8,200 |
| | | hsa-miR-203a | E2F1 | miR-SNORD48 | 223,320 | 0,270 | 0,016 | 1,000 | 0,001 | -9,702 |
| | | hsa-miR-205-5p | E2F1 | miR-SNORD48 | 311,740 | 0,270 | 0,011 | 1,000 | 0,001 | -10,117 |
| | | hsa-miR-221-3p | CDKN1C | miR-SNORD48 | 1,070 | 4,080 | 0,016 | 1,000 | 3,813 | 1,931 |
| | | hsa-miR-222-3p | CDKN1C | miR-SNORD48 | 1,390 | 3,540 | 0,048 | 1,000 | 2,546 | 1348,000 |
| | | hsa-miR-375 | TP53 | miR-SNORD48 | 3,460 | 1,980 | 0,015 | 1,000 | 0,572 | -0,806 |
| | 639 | hsa-miR-200a-3p | TP53 | miR-SNORD48 | 147,380 | 0,080 | 0,000 | 1,000 | 0,001 | -10,850 |
| | | hsa-miR-203a | E2F1 | miR-SNORD48 | 223,320 | 73,350 | 0,017 | 1,000 | 0,328 | -1,608 |
| | | hsa-miR-205-5p | E2F1 | miR-SNORD48 | 311,740 | 0,500 | 0,001 | 1,000 | 0,002 | -8,965 |
| | | hsa-miR-221-3p | CDKN1C | miR-SNORD48 | 1,070 | 2,910 | 0,024 | 1,000 | 2,719 | 1,443 |
| | | hsa-miR-222-3p | CDKN1C | miR-SNORD48 | 1,390 | 0,750 | 0,026 | 1,000 | 0,539 | -0,891 |
| | | hsa-miR-375 | TP53 | miR-SNORD48 | 3,460 | 3,090 | 0,192 | 1,000 | 0,893 | -0,163 |
| | 001 | hsa-miR-200a-3p | TP53 | miR-SNORD48 | 147,380 | 0,080 | 0,000 | 1,000 | 0,001 | -10,850 |
| | | hsa-miR-203a | E2F1 | miR-SNORD48 | 223,320 | 47,570 | 0,023 | 1,000 | 0,213 | -2,231 |
| | | hsa-miR-205-5p | E2F1 | miR-SNORD48 | 311,740 | 0,570 | 0,001 | 1,000 | 0,002 | -9,117 |
| | | hsa-miR-221-3p | CDKN1C | miR-SNORD48 | 1,070 | 2,980 | 0,011 | 1,000 | 2,785 | 1,477 |
| | | hsa-miR-222-3p | CDKN1C | miR-SNORD48 | 1,390 | 0,860 | 0,064 | 1,000 | 0,618 | -0,694 |
| | | hsa-miR-375 | TP53 | miR-SNORD48 | 3,460 | 3,550 | 0,689 | 1,000 | 1,171 | 0,227 |

Relative expression values were calculated as the miRNA amount of each gene relative to SNORD48 (used as reference) and normalized to the relative expression of normal control samples. 2^-DCt 2 numbers indicate mean expression values analysed by the 2^DDCt method obtained from three independent experiments in foetal thymuses and each tumour sample. Norm_1 and Norm_2 values represent normalized 2^-DCt 2 mean values. log2FC denotes the log-transformed (base 2) fold change normalized value of expression in each tumour sample relative to controls (foetal thymuses).
